# Improvement of Spontaneous Locomotor Activity in a Murine Model of Duchenne Muscular Dystrophy by N‐Acetylglucosamine Alone and in Combination With Prednisolone

**DOI:** 10.1096/fj.202500196R

**Published:** 2025-09-15

**Authors:** Masahiko. S. Satoh, Guillaume St‐Pierre, Ann Rancourt, Maude Fillion, Sachiko Sato

**Affiliations:** ^1^ Laboratory of DNA Damage Responses and Bioimaging, Research Centre of CHU de Quebec and Axis of Oncology, Faculty of Medicine Laval University Quebec Canada; ^2^ Glycobiology and Bioimaging Laboratory of Research Center for Infectious Diseases and Axis of Infectious and Immunological Diseases, Research Centre of CHU de Quebec, Faculty of Medicine Laval University Quebec Canada

## Abstract

N‐acetylglucosamine (GlcNAc) is an endogenous compound with intracellular concentration closely linked to the biosynthesis of acetyllactosamine‐rich N‐linked oligosaccharides. These oligosaccharides interact with mammalian lectin galectin‐3, mediating cell surface receptor dynamics as well as cell–cell and cell‐extracellular matrix interactions. Our previous and recent studies suggest that GlcNAc, in conjunction with galectin‐3, enhances muscle regeneration in vitro. We have also demonstrated that intraperitoneal GlcNAc administration improves muscle strength in *mdx* mice, a murine model of Duchenne muscular dystrophy (DMD). Here, we show that oral administration of GlcNAc significantly improves the spontaneous locomotor activity of *mdx* mice. Daily treatment with United States Pharmacopeia‐grade GlcNAc at doses of 0.6, 1.2, 1.8, and 2.4 g/kg body weight for 35 days significantly enhanced nocturnal spontaneous locomotor activity, with the 1.2 g/kg body weight dose (equivalent to 0.144 g/kg body weight in humans) reducing damages of extensor digitorum longus muscle by nearly 50%. Although consecutive forced exercises, specifically horizontal and downhill treadmill running, reduced GlcNAc‐mediated improvement, mice treated with 0.6 and 1.2 g/kg body weight still showed increased overall spontaneous locomotor activity under this condition, despite the lack of protection against repeated eccentric contraction‐induced injury. These findings suggest that GlcNAc enhances overall muscle health, possibly through mechanisms other than direct protection from muscle injury. One possible contributing mechanism may involve enhanced muscle repair or regeneration, as suggested by our related in vitro myogenesis work. Notably, co‐administration of GlcNAc with prednisolone, a corticosteroid commonly prescribed for DMD patients, further enhanced spontaneous locomotor improvement in *mdx* mice compared to prednisolone alone. These findings suggest that GlcNAc has the potential to improve the clinical status of DMD patients, either as a monotherapy or in combination with corticosteroids.

## Graphical abstract

Spontaneous locomotor activity of Duchenne muscular dystrophy (DMD) model mice was monitored using a cage system with external sensors. Mice received oral United State Pharmacopeia‐grade N‐acetylglucosamine (GlcNAc), prednisolone (Pred)—a current standard of care for DMD—or a combination of both. Treatment with GlcNAc as a monotherapy resulted in a significant increase in locomotor activity. Furthermore, the combination of GlcNAc and Pred produced the highest level of activity, demonstrating a significant additive therapeutic improvement for DMD.

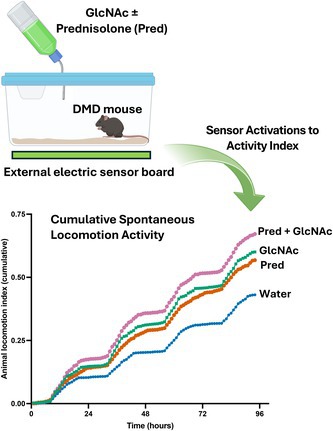

## 1 Introduction

Duchenne muscular dystrophy (DMD) is an inherited X‐linked disorder characterized by progressive muscle wasting, affecting approximately 1 in 3500 male births worldwide [1, 2, 3]. DMD is caused by mutations in the dystrophin gene, which encodes the dystrophin protein, a crucial component of the dystrophin‐associated glycoprotein complex (DAG) that connects the actin cytoskeleton to the extracellular matrix [3, 4, 5, 6, 7]. The absence or malfunction of dystrophin leads to the reduction of surface DAG, thereby compromising the integrity of muscle fibers, making them susceptible to damage and leading to chronic inflammation, fibrosis, and ultimately, muscle degeneration [1, 3, 5, 6, 7, 8, 9].

Corticosteroids, such as prednisolone, are commonly administered to DMD patients to reduce inflammation and slow disease progression, though they are associated with significant side effects [10, 11, 12]. Recent therapeutic approaches include gene therapy, exon skipping, and the development of novel corticosteroids like vamorolone and deflazacort, which aim to minimize side effects while preserving anti‐inflammatory efficacy [10, 11, 12]. Previously, we reported that intraperitoneal administration of N‐acetylglucosamine (GlcNAc) improved the muscular strength of *mdx* mice, a well‐established mouse model of Duchenne muscular dystrophy (DMD) [13]. This finding suggests that GlcNAc may have potential as a therapeutic agent for DMD patients, either as a standalone treatment or in combination with other DMD interventions. Given the necessity of daily administration, oral delivery of GlcNAc is particularly advantageous for children with DMD. However, it was previously unknown whether oral administration could effectively mitigate disease progression. In this study, we investigate the therapeutic potential of oral GlcNAc as a standalone treatment and in combination with prednisolone in *mdx* mice.

GlcNAc is an endogenous compound that serves as a precursor for UDP‐GlcNAc, which acts as a substrate for various N‐acetylglucosaminyltransferases involved in protein glycosylation. These oligosaccharide‐processing enzymes, including mannosyl glycoprotein N‐acetylglucosaminyltransferases (MGATs), play a crucial role in the biosynthesis of N‐linked oligosaccharides within the secretory pathway [14, 15, 16, 17]. The increase in intracellular GlcNAc and UDP‐GlcNAc, which can be augmented by GlcNAc administration, promotes the production of glycoproteins that carry acetyllactosamine‐rich oligosaccharides [16, 17]. These oligosaccharides play a critical role in the regulation of the dynamics of various membrane glycoproteins as well as cell–cell and cell–cell matrix interaction; this is partly mediated by galectin‐3, which specifically binds to those oligosaccharides [18, 19, 20, 21, 22, 23, 24, 25, 26]. Our previous and recent studies suggest that the interaction between oligosaccharide and galectin‐3 promotes myogenesis in vitro [13, 27]. Given that differentiating myoblasts and skeletal muscles of *mdx* mice and DMD patients express high levels of galectin‐3 [28, 29, 30], the interaction between the oligosaccharides and galectin‐3 is likely important in myogenesis.

Orally administered GlcNAc is rapidly absorbed in the upper gastrointestinal tract and enters the circulation as GlcNAc within 30 min, with clearance occurring within 2–3 h. Its bioactive form, UDP‐GlcNAc, accumulates in various organs including the liver, kidney, and spleen [17, 31, 32]. Previous preclinical toxicity studies in rats demonstrated that chronic oral administration of GlcNAc 2.5 g/kg body weight (BW) per day (equivalent to 0.6 g/kg BW per day in humans) for 52 weeks results in no adverse effects or histopathological changes in tissues [33, 34, 35]. Additionally, GlcNAc demonstrated safety in patients with inflammatory bowel disease and multiple sclerosis at doses of 6 and 12 g per day for 4 weeks with no reported adverse effects [36, 37]. Breastfed infants are naturally exposed to relatively high levels of GlcNAc, as this sugar is abundantly present in human milk oligosaccharides, with concentrations gradually decreasing from about 1.5 mg/mL at birth to 0.6 mg/mL by 13 weeks of age [38]. The infant gut microbiota has the capacity to break down these oligosaccharides and release GlcNAc as a free monosaccharide [39]. Based on average intake volumes, this translates to an estimated GlcNAc consumption of 0.5–1.5 g per day, or approximately 100–300 mg per kg of body weight in a 5‐kg infant. GlcNAc is a water‐soluble monosaccharide with a light, sweet taste, likely contributing to high patient acceptance of its oral administration if therapeutic efficacies are demonstrated.

Here we report that oral administration of GlcNAc for 35 days can improve the spontaneous locomotor activity of *mdx* mice. Oral GlcNAc administration of 0.6, 1.2, 1.8, and 2.4 g/kg BW (equivalent to doses of 72, 144, 216, and 288 mg/kg BW per day, respectively, in humans [35]) did not affect BW or muscle mass in *mdx* mice. Spontaneous locomotor activity in *mdx* mice improved significantly at all doses of GlcNAc, and muscle damage was reduced with the administration of 1.2 g/kg BW GlcNAc, while creatine phosphokinase levels remained unchanged. To determine whether GlcNAc treatment preserves muscle health against forced exercises, mice were subjected to consecutive forced exercises (horizontal and downhill treadmill running) during the final week of the 35‐day treatment. Although those exercises reduced GlcNAc‐promoted locomotor activity, treatment with 0.6 and 1.2 g/kg BW still increased locomotor activity even after forced exercises. Moreover, co‐administration of GlcNAc with prednisolone further enhanced nocturnal locomotor activity. These findings suggest that while the optimal dose of GlcNAc for DMD patients remains to be determined, GlcNAc could be a promising therapeutic agent for improving muscle function in DMD, either as a standalone treatment or in combination with other interventions.

## 2 Materials and Methods

### 2.1 Animal Studies

All animal experiments were conducted in accordance with the policies of the Comités de Protection des Animaux at Université Laval (CPAUL‐3). Male *mdx* dystrophic mice (C57BL/10ScSn‐Dmd^*mdx*^/J) were purchased from The Jackson Laboratory (Bar Harbor, ME, USA) and bred at the CRCHU animal facility. The mice were housed under controlled conditions: temperature (23°C ± 2°C), humidity (50% ± 5%), and light (12:12 h light–dark cycle). They had unlimited access to food and water. GlcNAc (2.4, 4.8, 7.2, and 9.6 mg/mL) and/or prednisolone (1 mg/kg BW per day, based on a 5 mL daily water intake) were administered orally via voluntary intake through their drinking water to the *mdx* mice. Water consumption was routinely confirmed three times per week. There was no significant difference in the volume of water consumed by GlcNAc‐treated mice compared to the control group. All treatments started at the age of 3 weeks, and the treatment lasted for 35 ± 1 days.

### 2.2 Protocols

#### 2.2.1 Protocol 1

GlcNAc was orally administered through drinking water at doses of 0 (control), 2.4, 4.8, and 9.6 mg/mL for 35 days. Mice were sacrificed for the examination of muscles and serum CPK levels.

#### 2.2.2 Protocol 2

GlcNAc was orally administered through drinking water at doses of 0, 2.4, 4.8, 7.2, and 9.6 mg/mL for the entire 35 days. After 26 ± 1 days of treatment, the mice were subjected to treadmill exercises (see below for the detailed conditions, Figure 3A). Spontaneous locomotor activity for the last 6.5 days was monitored using the DVC system. Twenty‐four hours before their sacrifice, mice were injected with Evans Blue Dye (EBD).

#### 2.2.3 Protocol 3

Mice were orally administered GlcNAc at doses of 0, 2.4, 4.8, 7.2, and 9.6 mg/mL, with or without prednisolone for 35 ± 1 days. Spontaneous locomotor activity for the last 3.5 days was monitored using the DVC system.

### 2.3 Materials

United States Pharmacopeia (USP)‐grade GlcNAc (100% pure: Ultimate Glucosamine), was obtained from Wellesley Therapeutics, Ontario, Canada. Prednisolone was purchased from Abcam.

### 2.4 Muscle Weights

Following sacrifice, tibialis anterior (TA), extensor digitorum longus (EDL), and soleus (Sol) muscles were dissected and weighed. To account for differences in animal size, muscle mass was normalized to total BW.

This method was considered appropriate for the current study as no significant treatment‐induced alterations in BW gain were observed in our primary experimental cohorts. Furthermore, while normalization to a skeletal measure such as tibia length is also utilized [40].

### 2.5 CPK Activity Assay

Blood samples were centrifuged to separate the supernatant after incubation at room temperature. Serum CPK assays were performed using the Pointe Scientific Creatine Kinase Liquid Reagent Set, following the manufacturer's protocol. Serum (5 μL) was incubated with 100 μL of the working reagent at 37°C. Dye absorbance was measured spectrophotometrically at 340 nm over a period of 5 min. The values were calculated according to the manufacturer's instructions, and the data were expressed as units per liter (CPK U/L).

### 2.6 Muscle EBD Uptake Experiments

Muscle damage was assessed using the penetration of Evance blue dye (EBD; Sigma‐Aldrich) into muscles following the method described by Wooddell et al. [41] Briefly, EBD was dissolved in phosphate‐buffered saline at a concentration of 10 mg/mL and injected intraperitoneally at a dose of 100 μL per 10 g of BW. Twenty‐four hours after the injection, the mice were sacrificed, and the TA muscles were harvested and frozen at −80°C. The frozen TA muscles were then ground into a powder, and 50 mg of the powder was used for quantification. *N*,*N*‐Dimethylformamide (1 mL) was added to the muscle powder, followed by incubation for 24 h at room temperature. After centrifugation at 6500 ***g*** for 25 min, the supernatant was collected, and the absorbance was measured at 630 nm to evaluate the extent of muscle damage.

### 2.7 Histological Analysis

Muscles were dissected and frozen in Tissue‐Plus O.C.T. Compound (Fisher Healthcare) using isopentane cooled with liquid nitrogen. Frozen muscle sections (8–10 μm) were fixed in cold acetone or 4% paraformaldehyde. H&E staining of EDL muscles was performed following standard protocols. We chose the EDL muscles as they are composed almost exclusively of fast‐twitch fibers, which are more susceptible to eccentric contraction‐induced damage during daily activity and downhill treadmill exercise. Images of the H&E‐stained muscle sections were captured using a standard microscope equipped with a color camera. The images were then converted to grayscale and segmented using in‐house software available on GitHub (DOI https://doi.org/10.5281/zenodo.12988726) [42]. Segmented images were used to quantify muscle area, muscular fiber size, inflammatory area, and matrix space. Quantification was performed with manual correction as needed. The percentages of inflammatory area and matrix space were then calculated.

### 2.8 Treadmill Performance Tests

The treadmill performance test was conducted using a motor‐driven MK‐680S treadmill system (Columbus Instruments) to evaluate the muscle performance of mice after 3 weeks of housing. On Day 1, the mice were placed on the treadmill and ran for 10 min at a speed of 5 m/min on a flat surface. After a rest day on Day 2, the mice ran again on Day 3 for 10 min at an increased speed of 10 m/min. Following another rest day on Day 4, a third run was performed on Day 5 for 10 min at a speed of 15 m/min on a flat treadmill. After two more rest days on Day 6 and Day 7, the final treadmill run was conducted on Day 8 on a 15° downhill slope, following this protocol: 2 min at 8 m/min, 2 min at 10 m/min, 5 min at 12 m/min, 5 min at 13 m/min, and 5 min at 14 m/min. During the last 30 min, the run was conducted at 15 m/min, for a total duration of 49 min. Exhaustion was defined as the mouse stopping running for 5 consecutive seconds without attempting to resume running. If a mouse reached exhaustion, it was given a 2‐min rest before resuming the downhill treadmill run. As a final exclusion criterion, if the same mouse reached exhaustion for a second time, it was returned to its cage.

### 2.9 Locomotor Activity Measurement

The locomotor activity of mice was measured using the Digital Ventilated Cage (DVC) Pre system (Tecniplast, Italy), which is equipped with 12 electrodes. One mouse was housed in each cage under a 12:12‐h light: dark cycle. Locomotor activity was continuously monitored, and the data were stored using the DVC system's associated data storage. The collected data were then analyzed using Prism GraphPad Software.

### 2.10 Statistical Analysis

Statistical significance was determined with one‐way ANOVA and two‐way ANOVA (the significances are explained in the figure legends). All statistical analyses were performed with Prism (GraphPad Software, La Jolla, CA, USA), and differences were considered significant at *p* < 0.05.

## 3 Results

### 3.1 Effect of GlcNAc on BW, Muscle Mass, and Creatine Phosphokinase Release (Protocol 1)

**FIGURE 1:**
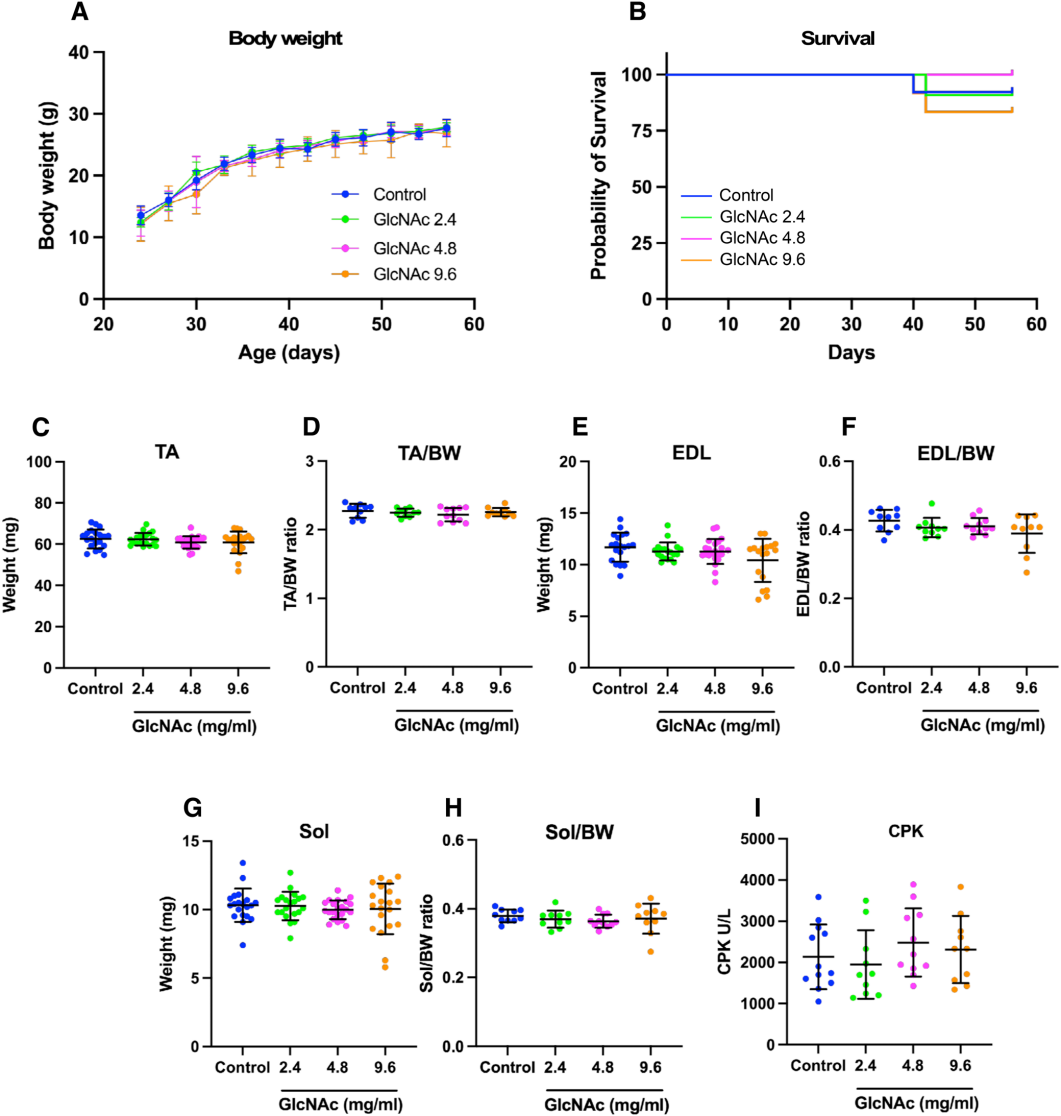
Impact of GlcNAc on BW, survival, and muscle mass in *mdx* mice (Protocol 1). GlcNAc (2.4, 4.8, and 9.6 mg/mL) was administered orally via voluntary intake to *mdx* mice. Mice in the control group were provided with standard drinking water that did not contain any GlcNAc. (A) Mice were housed for 35 days, during which their BW was regularly measured. (B) The survival rate of the mice was monitored throughout the study. For both (A and B) the number of mice used was 10. (C–H) On day 35, the *mdx* mice were sacrificed, and the mass of the TA (C), TA mass relative to BW (D), EDL mass (E), EDL mass relative to BW (F), Sol mass (G), and Sol mass relative to BW (H) were measured. For (C, E, and G) the sample size was 20, and for D, F, and H, the sample size was 10. (I) CPK levels in the serum were also measured (*n* = 10). Statistical analyses were performed using ordinary one‐way ANOVA with Tukey's post hoc test (A and C–I), and Mantel‐Cox test (B). No significant differences were observed. (A and C–I) Data represent means ± standard deviations.

To evaluate the effects of GlcNAc on *mdx* mice, mice received United States Pharmacopeia (USP)‐grade GlcNAc continuously through their drinking water at the concentrations of 2.4, 4.8, and 9.6 mg/mL, which is equivalent to 0.6, 1.2, and 2.4 g/kg BW per day, respectively. Mice in the control group were provided with standard drinking water that did not contain any GlcNAc, serving as a baseline for comparison against the experimental groups. Over the course of 35 days, no significant differences were observed in BW (Figure 1A) or mortality (Figure 1B), indicating that GlcNAc administration does not adversely affect the growth or survival of *mdx* mice. After 35 days, the masses of the Tibialis Anterior (TA), Extensor Digitorum Longus (EDL), and Soleus (Sol) muscles were measured. No significant differences were found in the absolute mass of the TA (Figure 1C) or in the TA mass relative to BW (Figure 1D) between treatment groups. Similarly, no significant changes were observed in the absolute or relative masses of the EDL (Figure 1E,F) and Sol (Figure 1G,H) muscles. These data suggest that GlcNAc does not influence muscle weight. Additionally, while muscle damage in *mdx* mice is indirectly correlated with elevated plasma levels of creatine phosphokinase (CPK) [43, 44], GlcNAc treatment did not alter these levels (Figure 1I), suggesting that GlcNAc does not prevent the formation of muscle damage under basal conditions.

### 3.2 Histological Analysis of EDL

**FIGURE 2:**
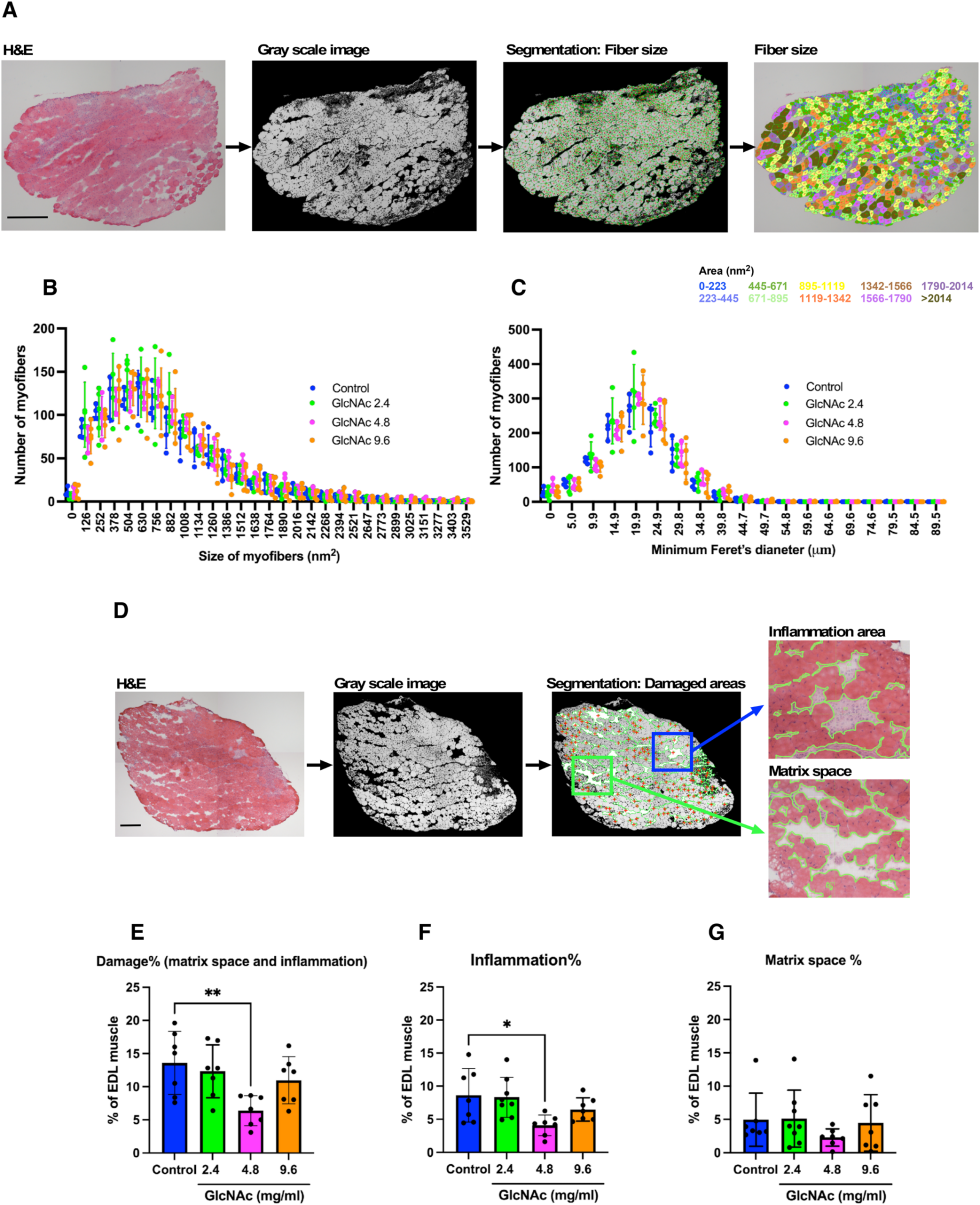
Effect of GlcNAc on muscular damage formation in *mdx* mice (Protocol 1). (A) Tissue sections of the EDL muscle were prepared and stained with H&E. Images were captured and converted to grayscale for segmentation analysis using in‐house software to measure muscle fiber sizes, which were illustrated using a color‐coding system. (B and C) The effect of GlcNAc on the muscle fiber size (B) and minimum Feret's diameter (C) of EDL was analyzed using in‐house software. Statistical analysis was performed using two‐way ANOVA, and no significant differences were observed. Data represent means ± standard deviations, with a sample size of 4 or 5 for each group. (D) Images of the EDL sections stained with H&E were captured and converted to grayscale for segmentation analysis using in‐house software to measure the areas of inflammation and matrix space. The detected areas used for quantification are indicated by green lines. (E–G) The percentage of damaged area was calculated by combining the inflammation area and matrix space, normalized to the total area of the EDL. The percentage of the inflammation area (F) and matrix space (G) was calculated by normalizing these areas to the total area of the EDL. Statistical analysis was performed using one‐way ANOVA with Dunnett's test. Significance levels are indicated as *p < 0.01, and **p < 0.005. Data represent means ± standard deviations, with a sample size of 7 for each group. (A and C) Scale bars indicate 100 nm.

Next, we analyzed the muscle fiber size of EDL using Hematoxylin–Eosin (H&E) stained sections. We focused on the EDL because it consists almost exclusively of fast‐twitch fibers, a fiber type that is particularly susceptible to damage from eccentric contractions. To objectively assess muscle fiber size, we employed our in‐house software, which converts H&E‐stained images into grayscale, performs image segmentation to identify muscle fibers, and quantifies their cross‐sectional areas across the entire muscle section, rather than relying on randomly selected fields of view (Figure 2A). Based on their size, muscle fibers were color‐coded, as illustrated in Figure 2A. Using this software, we investigated the effect of GlcNAc on muscle fibers of the entire muscle section (both size and minimum Feret's diameter) but found no significant differences (Figure 2B,C). We then quantitatively evaluated the matrix space and inflammatory areas using the same software, which converts images to grayscale and performs image segmentation to identify those areas. The software quantifies their sectional areas across the entire muscle section, as histological damage and inflammation area in *mdx* muscle samples vary substantially between animals even under identical treatment conditions, likely reflecting differences in the timing of muscle injury and regeneration cycles at the time of sacrifice (Figure 2D). Combined analysis of the matrix space and inflammatory sites revealed that *mdx* mice treated with 4.8 mg/mL GlcNAc exhibited significantly less damage (~50%) in the EDL muscles compared to the untreated control group (Figure 2E). The area of inflammation was also reduced (Figure 2F), although no significant reduction was observed in the matrix space (Figure 2G). This reduction in muscle damage occurred despite the lack of significant changes in CPK levels (Figure 1I). Given that our previous studies indicate that GlcNAc augments myogenesis in vitro [13, 27], the present results may imply that GlcNAc administration supports muscle recovery rather than preventing the initial onset of muscle damage.

### 3.3 Treadmill Exercise in *mdx* Mice (Protocol 2)

To further assess whether GlcNAc influences the initial onset of muscle damage and tests additional GlcNAc doses, we implemented Protocol 2 in which *mdx* mice were treated for 35 days with GlcNAc at concentrations of 2.4, 4.8, 7.2, and 9.6 mg/mL and subjected to downhill treadmill running. This forced exercise paradigm induces a uniform and repeatable eccentric contraction challenge, thereby standardizing the timing and extent of damage across animals. As such, it allows us to examine whether 35‐day GlcNAc treatment alters muscle susceptibility to contraction‐induced injury.

**FIGURE 3:**
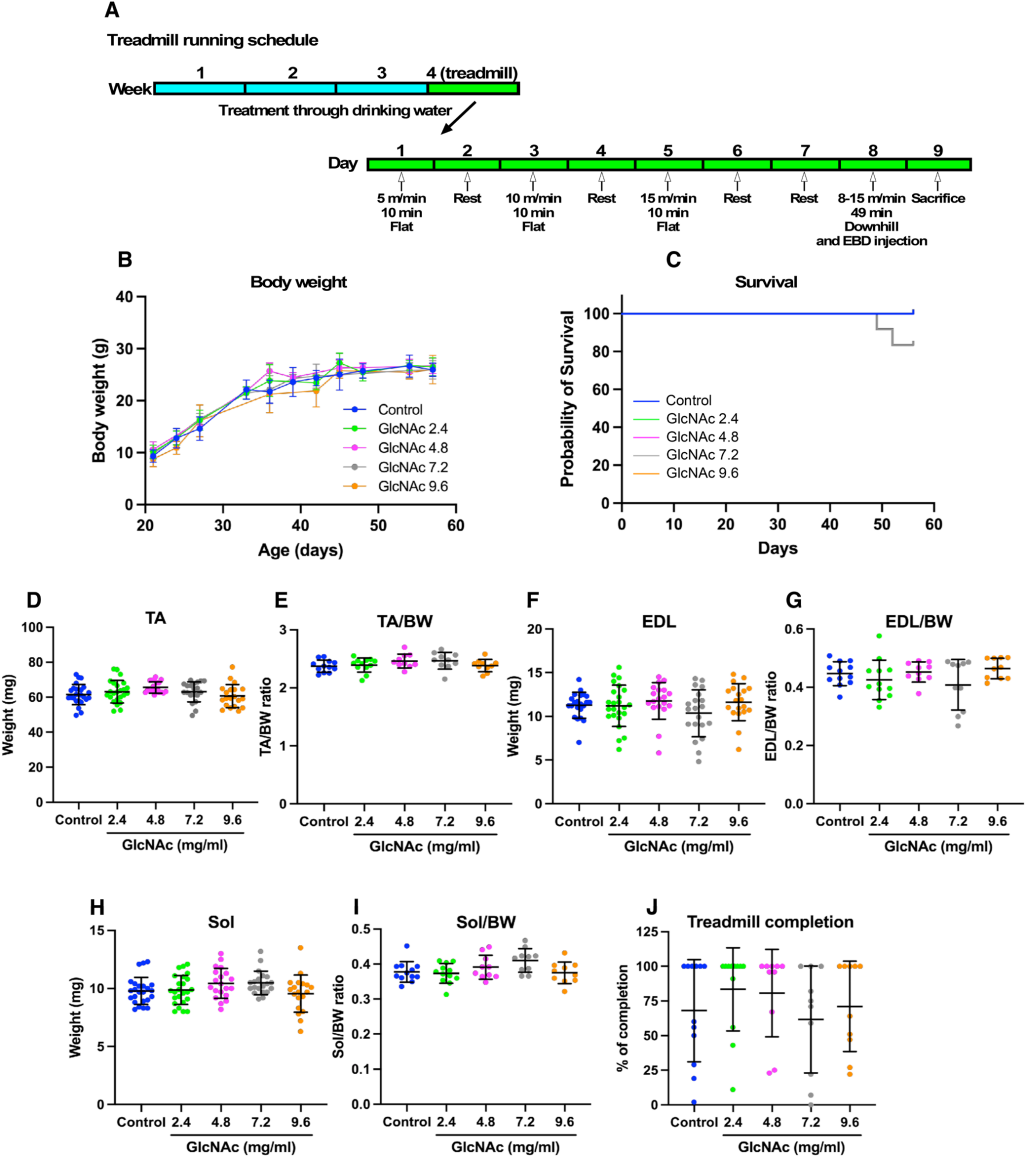
Impact of GlcNAc on BW, Survival, and Muscle Mass in *mdx* Mice Subjected to Treadmill Running (Protocol 2). (A) Treadmill exercise schedule. After 26 ± 1 days of treatment, *mdx* mice were acclimated to treadmill running with three sessions on a flat treadmill, as illustrated. On Day 8, the mice were subjected to a faster treadmill run on a 15° downhill incline, with speeds gradually increasing from 8 to 15 m/min over 49 min. Immediately after the run, EBD was injected to stain damaged muscles, and the mice were sacrificed the following day. (B) Mice's BW was regularly measured throughout the study. (C) The survival rate of the mice was monitored. For both panels (B and C), the number of mice in the Control, 2.4, 4.8, 7.2, and 9.6 mg/mL GlcNAc‐treated groups were 13, 14, 10, 11, and 10, respectively. (D–I) On the day following the treadmill run (Day 8), mice were sacrificed, and the mass of the TA (D), TA mass relative to BW (E), EDL mass (F), EDL mass relative to BW (G), soleus (Sol) mass (H), and Sol mass relative to BW (I) were measured. For panels (D, F, and H), the sample size was 20–24, and for panels (E, G, and I), the sample size was 10–12. (J) The percentage of mice that completed the treadmill run performed on Day 8 was calculated (sample size: 10–12). Statistical analyses were performed using ordinary one‐way ANOVA with Tukey's post hoc test (B and D–J) and the Mantel‐Cox test (C). No significant differences were observed. Data in panels (B and D–J) represent means ± standard deviations.

Three weeks after the initiation of GlcNAc administration, mice treadmill session began in the 4th week (Figure 3A). To acclimate the mice to treadmill running, training sessions were conducted on a flat treadmill at a speed of 5 m/min for 10 min on Day 1. After a day of rest, the speed was increased to 10 m/min for 10 min on Day 3. Following another day of rest, the speed was further increased to 15 m/min on Day 5. After two more days of rest, the final treadmill run was performed on Day 8, with a gradual increase in speed from 8 to 15 m/min over 49 min on a 15° downhill incline. After the run, Evans Blue Dye (EBD) was injected, and the mice were sacrificed the following day. No significant differences in BW (Figure 3B) or survival (Figure 3C) were observed between groups, and no significant differences were observed in the TA (Figure 3D,E), EDL (Figure 3F,G), or Sol (Figure 3H,I) muscle masses, whether absolute or relative to BW. To determine if the strenuous downhill protocol led to different drop‐out rates between groups, we quantified the percentage of mice that successfully completed the entire 49‐min run. A high completion rate was observed even in the untreated control *mdx* group, and there were no significant differences in this rate between the control and any of the GlcNAc‐treated groups (Figure 3J). This indicates that while the task was demanding, GlcNAc administration did not adversely affect the ability of the mice to tolerate the forced running exercise.

### 3.4 Muscle Damage Induced by Treadmill Running (Protocol 2)

**FIGURE 4:**
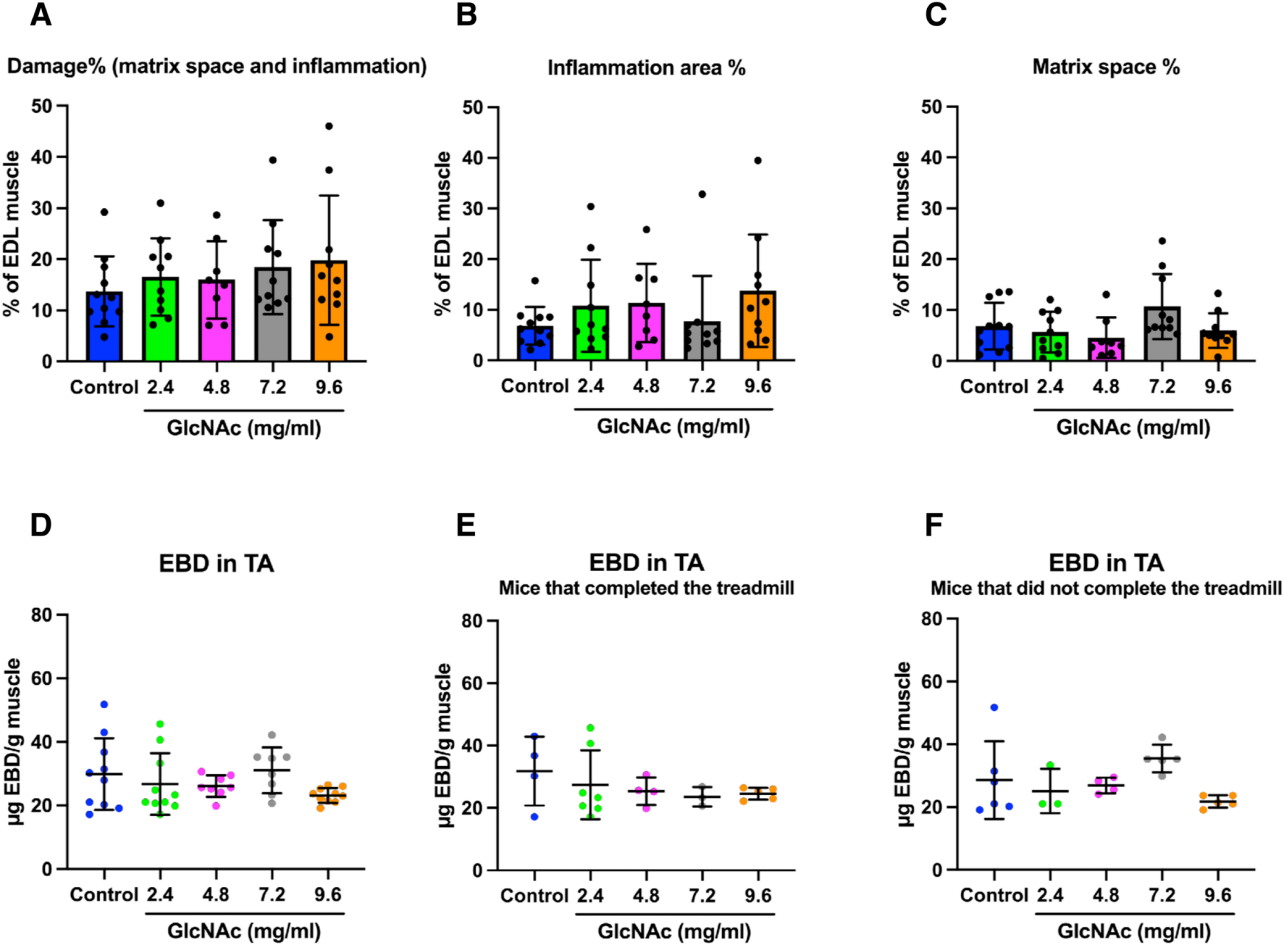
Effect of GlcNAc on Muscle Damage Formation in *mdx* Mice Subjected to Treadmill Running (Protocol 2). (A–C) Tissue sections of the EDL muscle were prepared and stained with H&E. Images were captured and converted to grayscale for segmentation analysis using in‐house software to distinguish between the inflammation area and matrix space. The percentage of the damaged area was calculated by combining the inflammation area and matrix space, normalized to the total area of the EDL (A). The percentage of the inflammation area (B) and matrix space (C) was calculated by normalizing these areas to the total EDL area. The sample size for panels (A–C) was 8–11. (D–F) Muscles were stained with EBD, which specifically marks damaged muscle areas. The dye was then extracted to determine its concentration. Analysis was performed on all mice, including those that completed and did not complete the treadmill running on Day 8 (D), only those mice that completed the run (E), and only those that did not complete the run (F). The sample size for panel (D) was 10–11, and for panels (E and F) it was 3–7. Statistical analysis was performed using one‐way ANOVA with Tukey's post hoc test. No significant differences were observed. Data represent means ± standard deviations.

Quantitative analysis of damage sites using H&E‐stained sections of the EDL muscle following treadmill running revealed that muscle damage occurred across all GlcNAc‐treated groups, with no significant differences from non‐treated mice (Figure 4A–C). To further quantify muscle damage, EBD, which penetrates damaged myofiber, was injected immediately after the Day 8 treadmill run (Figure 3A), and the mice were sacrificed the following day. The TA muscle was then used to extract and quantify the concentration of EBD, assessing the extent of muscle damage. No statistically significant differences were found when analyzing all mice (Figure 4D), as well as when analyzing mice that completed (Figure 4E) or did not complete (Figure 4F) the running separately. Taken together with the CPK data (Figure 1I) and the results shown in Figure 2D,E, which indicated significantly less damage in mice treated with 4.8 mg/mL GlcNAc without any forced exercise, these findings suggest that while GlcNAc does not prevent muscle damage formation induced by forced downhill exercises (repeated eccentric contractions), it exerts positive effects on the muscle health of *mdx* mice, particularly at a concentration of 4.8 mg/mL GlcNAc.

### 3.5 Analysis of the Spontaneous Locomotor Activity of *mdx* Mice

**FIGURE 5:**
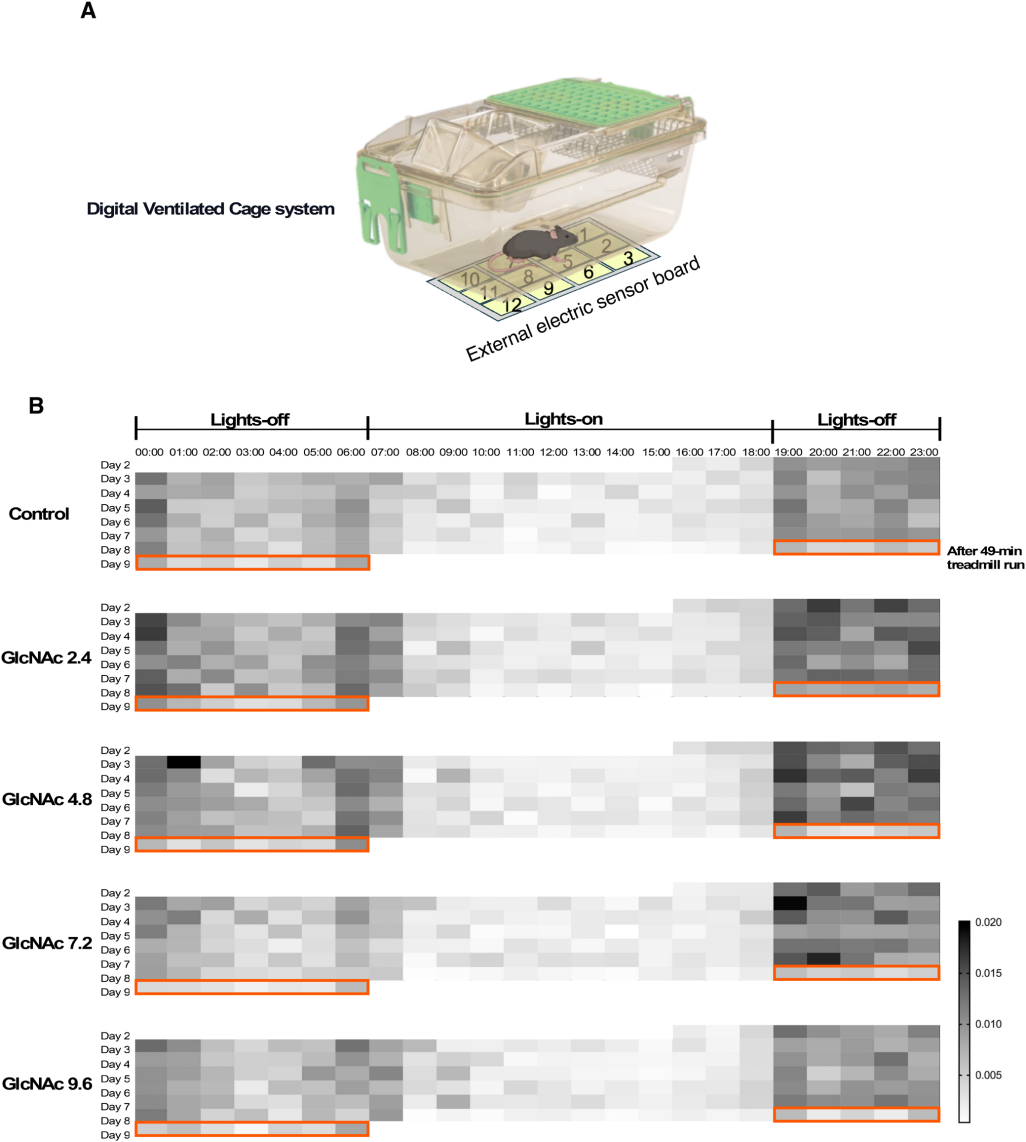
Overview of Spontaneous Locomotor Activity Analysis in *mdx* Mice (Protocol 2). (A) Spontaneous activity of *mdx* mice was monitored using the Digital Ventilated Cage (DVC) system. The system utilizes twelve electrode at the bottom of each cage, generating a low‐voltage electromagnetic field. The presence and movement of a mouse alter the capacitance of this field. An “activation” event is registered whenever this capacitance change exceeds a pre‐set threshold, with raw data captured four times per second. This sensitive detection method allows for the quantification of not only ambulatory movements (locomotion) but also subtle, non‐ambulatory activities such as grooming and turning. (B) An example of locomotor activity measured over 6.5 days is illustrated. This example follows the schedule shown in Figure 3A, which outlines the treadmill run. Locomotor activity measurement began on Day 2, the resting day after the first treadmill run. The activity is represented by a heatmap, with colors ranging from light gray (low activity) to dark gray (high activity). The data depicts hourly locomotor activity, with lights‐off and lights‐on periods marked. On Day 8, a 49‐min run was performed, and the orange box highlights the subsequent lights‐off period, during which the mice's locomotor activity was reduced.

The analysis of muscle damage through histological techniques can be affected by various factors, including the timing of damage formation. For example, significant damage may be detected if it occurs shortly before the sacrifice of a mouse, whereas earlier damage that has been partially repaired and regenerated may appear less severe. This variability is likely influenced by the autonomous behavior and continuous spontaneous locomotor activity of the mice. To minimize the influence of such ambiguous factors, it is essential to monitor the spontaneous locomotor activity of *mdx* mice continuously under conditions where no significant artificial stress is applied. For this purpose, we utilized the Digital Ventilated Cage (DVC) system, which employs 12 electrode sensors positioned at the bottom of the cage (Figure 5A) [40, 45]. Electromagnetic field lines are generated between the electrodes, and the presence of the mice disrupts these lines, allowing the system to detect their location and measure locomotor activity. This system allows for continuous, stress‐free monitoring of spontaneous locomotor activity, providing insights into how GlcNAc affects the muscles involved in locomotion.

The mouse was housed individually during the period of locomotor activity monitoring. Figure 5B illustrates representative results of the average hourly locomotor activity of the mice, with higher activity levels shown in dark gray and lower activity levels in light gray on a heat map. During the lights‐off period (12 h), the mice exhibited higher locomotor activity, consistent with their nocturnal nature. The DVC system, therefore, accurately captures the natural circadian rhythm of the mice, reflecting their typical locomotor activity.

For our primary endpoint, we utilized a cumulative spontaneous locomotion index derived from activation density. We selected this metric over traditional distance traveled because it provides a more comprehensive assessment of overall motor function in a naturalistic setting. Activation density is a validated metric that quantifies movement based on changes in electrical capacitance measured by an array of 12 electrodes beneath the cage floor. An “activation” event is registered whenever an animal's proximity or movement causes a capacitance change exceeding a set threshold. The raw data for these activation events were captured four times per second [45]. The key advantage of this metric is its ability to capture a broader spectrum of physiologically relevant behaviors. While measures like distance and speed are effective for assessing directed travel, activation density also registers subtle, non‐ambulatory movements such as grooming and turning. The original validation study notes that this metric accounts for any activity occurring near an electrode, not just linear displacement [45]. These fine motor actions require complex skeletal muscle coordination, making activation density an inherently more sensitive measure in the context of neuromuscular disease. The validation study confirms that while activation density strongly correlates with video‐tracked distance (average Pearson's *R* = 0.9323), its unique sensitivity to local movements provides a more complete representation of functional capacity in *mdx* mice [45].

### 3.6 Impact of GlcNAc on the Spontaneous Locomotor Activity of *mdx* Mice (Protocol 3)

**FIGURE 6:**
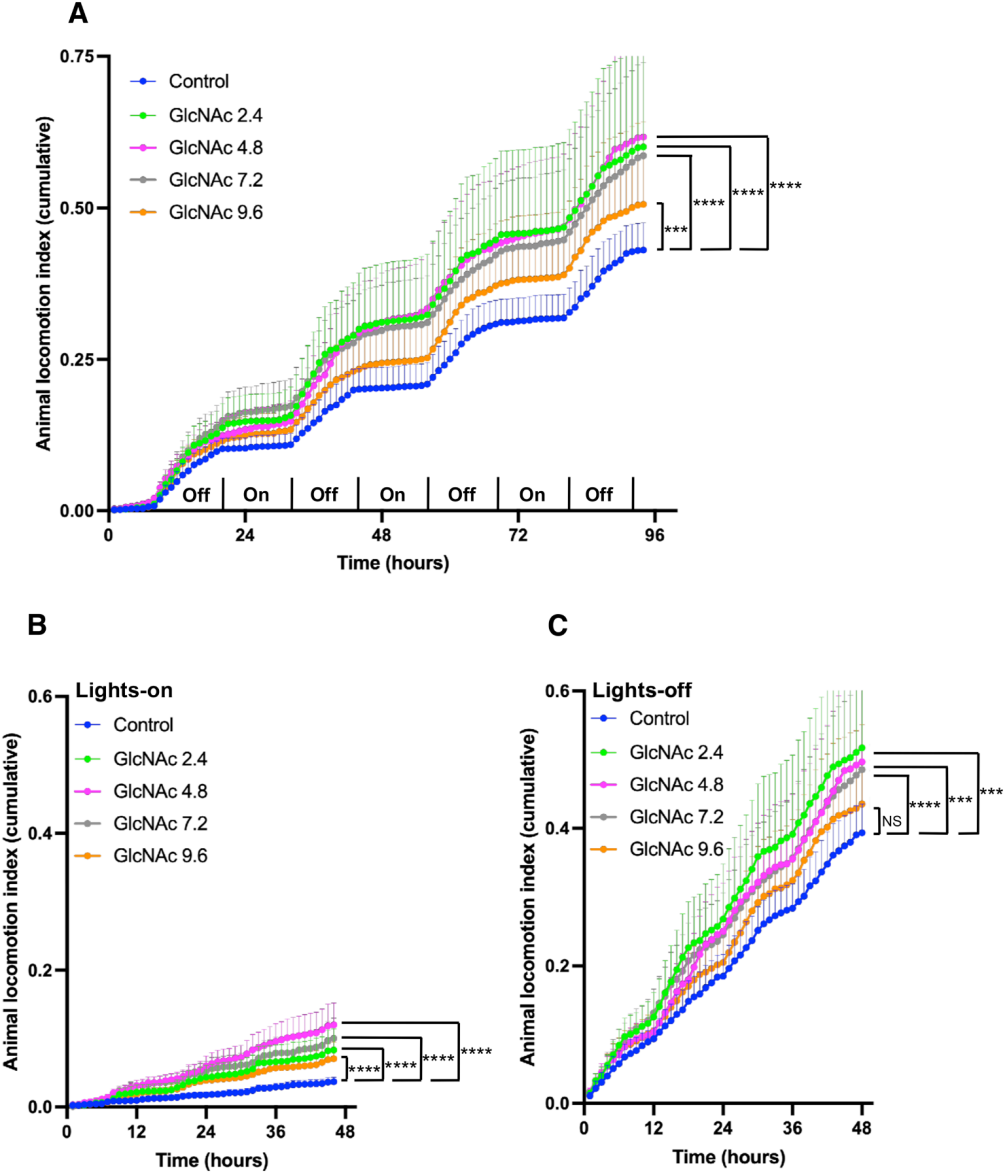
Effect of GlcNAc on the Spontaneous Locomotor Activity Index of *mdx* Mice (Protocol 3). Mice were treated with the indicated doses of GlcNAc for 35 days, and locomotor activity was measured over the final 3.5 days. The activity index was derived from raw data captured at 4 Hz, binned into 1‐s intervals, and then summed hourly for plotting. Error bands represent the SEM. For all panels, treatment groups are indicated by color as follows: Control non‐treated (blue), GlcNAc 2.4 (green), GlcNAc 4.8 (magenta), GlcNAc 7.2 (gray), and GlcNAc 9.6 (orange). (A) Cumulative locomotor activity over the full 84‐h monitoring period. (B) Cumulative locomotor activity during the lights‐on periods only. (C) Cumulative locomotor activity during the lights‐off periods only. Statistical analysis was performed using two‐way ANOVA with Dunnett's test. Significance levels are indicated as ***p < 0.001 and ****p < 0.0001, with “NS” denoting no significance. The number of mice used for Control (0, untreated), and for 2.4, 4.8, 7.2, and 9.6 mg/mL GlcNAc groups were 4, 5, 6, 5, and 6, respectively.

In Protocol 3, we measured the spontaneous locomotor activity index of *mdx* mice for 1 week following 3 weeks of GlcNAc treatment at concentrations ranging from 0 (control) to 9.6 mg/mL (Figure 6A). Control data related to BW, survival, and the mass of TA, DEL, and Sol muscles are shown in Figure S1, with no significant differences observed. While Figure 5B illustrates hourly average locomotor activity in a heat map format, Figure 6A shows the cumulative locomotor activity index over the monitoring period. The flat phase represents the locomotor activity during the lights‐on period, with increased activity during the lights‐off period (Figure 6A). In this primary analysis, mice receiving 2.4, 4.8, 7.2, and 9.6 mg/mL showed significantly higher spontaneous locomotor activity compared to the untreated control group. The activity of mice treated with 9.6 mg/mL was still higher than the control group, but lower than those treated with 2.4, 4.8, and 7.2 mg/mL.

To further probe the nature of these activity patterns, we performed a supplementary high‐resolution analysis using the raw, unbinned data (0.25‐s intervals), which is more sensitive to brief, fine‐scale movements. This analysis confirmed that all treatment groups were significantly more active than the non‐treated controls. However, it revealed a more defined dose–response peak, with the highest activity observed in the 4.8 mg/mL group, followed in descending order by the 2.4 and 7.2 mg/mL groups, which showed similar intermediate effects (see Figure S2A). The 9.6 mg/mL group showed the least benefit. Taken together, these findings suggest that while all tested doses are effective, there is an optimal GlcNAc concentration range for promoting overall spontaneous activity in *mdx* mice.

To understand the nature of this increased activity, we analyzed the diurnal (lights‐on) and nocturnal (lights‐off) periods separately (Figure 6B,C). GlcNAc treatment led to a significant increase in total locomotor activity during both the active, lights‐off period (Figure 6C) and the typically inactive, lights‐on period (Figure 6B). A more detailed temporal analysis revealed that the increased activity during the lights‐on phase was most pronounced during the first hour immediately following the transition from dark to light (see heatmap, Figure 5B).

Consistent with the activity index data, analysis of the total distance traveled showed that GlcNAc treatment increased movement (Figure S2B). The most significant increases in cumulative distance were observed in the 4.8 and 7.2 mg/mL groups. The 2.4 mg/mL group also showed a significant increase, while the distance traveled by the 9.6 mg/mL group was not significantly different from the control group.

Histological analysis of muscle damage (Figure 2) suggests that only 4.8 mg/mL GlcNAc showed a significant effect in reducing damage formation. However, these locomotor activity results indicate that various doses of GlcNAc, particularly 2.4, 4.8, 7.2, and 9.6 mg/mL, have a significant effect on enhancing the locomotor activity of *mdx* mice across different times of the day. Additionally, these doses of GlcNAc did not show any effect on BW, survival rate, or the mass of TA, EDL, and Sol muscles (Figure S1), suggesting that the promoted locomotor activity did not have any harmful effects on the *mdx* mice.

### 3.7 Impact of GlcNAc on the Spontaneous Locomotor Activity of *mdx* Mice Subjected to Treadmill Running (Protocol 2)

In Protocol 2, we tested if GlcNAc treatment could improve functional outcomes in *mdx* mice following contraction‐induced injury. Our rationale was that by subjecting all mice to a standardized eccentric contraction challenge (downhill treadmill running), we could induce a more uniform level of muscle damage. This allows for a clearer assessment of whether the 35‐day GlcNAc treatment improved functional performance after an injury challenge.

**FIGURE 7:**
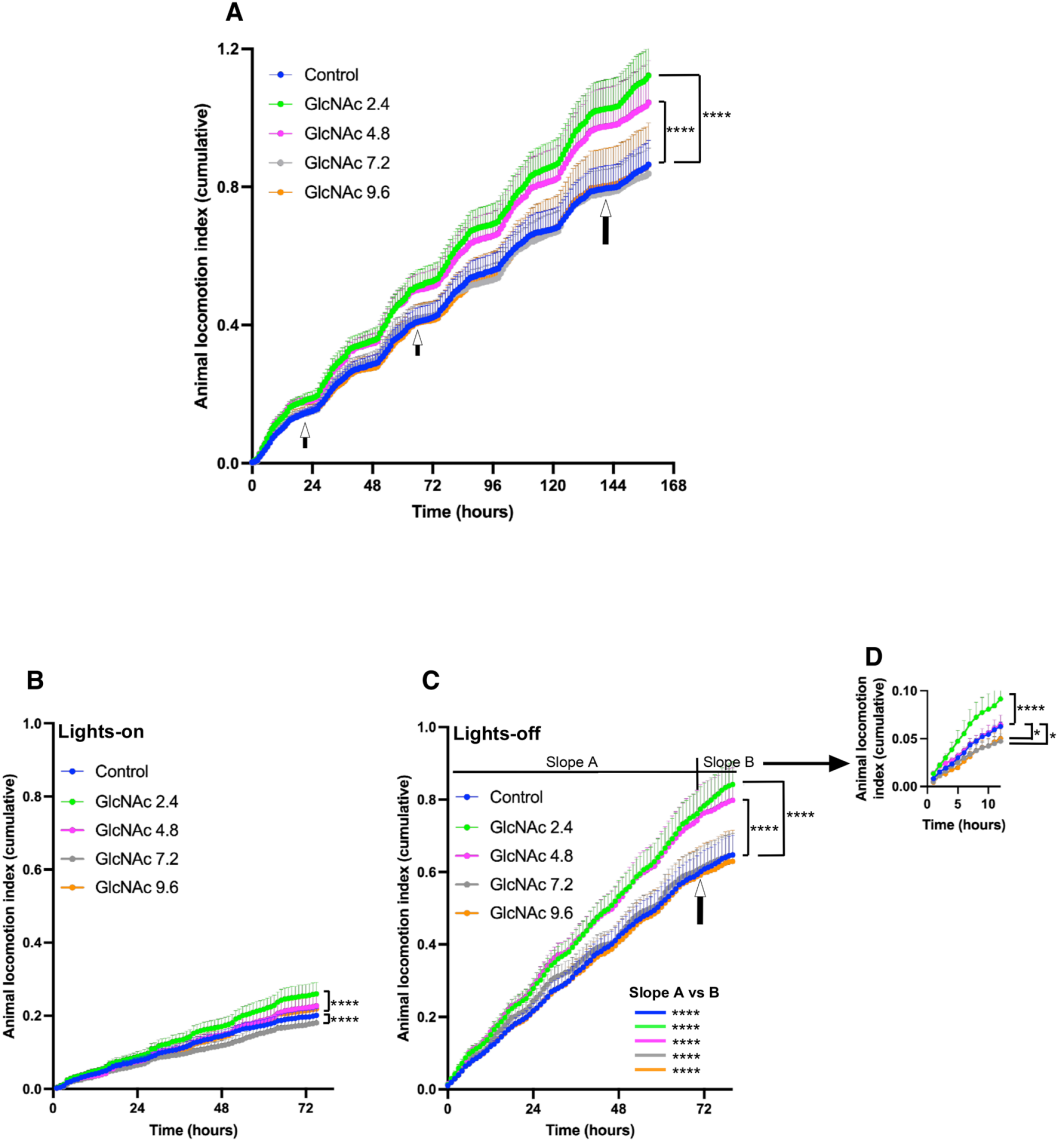
Effect of GlcNAc on the Spontaneous Locomotor Activity Index of *mdx* Mice Subjected to Treadmill Running (Protocol 2). Mice were treated with or without GlcNAc for 35 days. In the last week of the treatment, mice were subject to treadmill running according to the schedule illustrated in Figure 3A. Locomotor activity was measured over the final 6.5 days before the mice were sacrificed. The activity index was derived from raw data captured at 4 Hz, binned into 1‐s intervals, and then summed hourly for plotting. Error bands represent the SEM. For all panels, treatment groups are indicated by color as follows: Control non‐treated (blue), GlcNAc 2.4 (green), GlcNAc 4.8 (magenta), GlcNAc 7.2 (gray), and GlcNAc 9.6 (orange). (A) Cumulative locomotor activity index during both lights‐on and lights‐off periods. The first and second small arrows correspond to the acclimation treadmill runs performed on Day 3 and Day 5, respectively (Figure 3A). The large arrow indicates when the 49‐min treadmill run with a 15° downhill incline, gradually increasing in speed from 8 to 15 m/min, was conducted. (B) Cumulative locomotor activity index during the lights‐on period. (C) Cumulative locomotor activity index during the lights‐off period. The arrow marks the time of the 49‐min treadmill run. “Slope A” and “Slope B” refer to the periods before and after the run, respectively. (D) Cumulative locomotor activity index after the 49‐min treadmill run. Statistical analysis was performed using two‐way ANOVA with Dunnett's test. For the analysis of Slope A and Slope B, two‐way ANOVA with Tukey's test was used. Significance levels are indicated as *p < 0.05 and ****p < 0.0001. The number of mice used in the Control group and the 2.4, 4.8, 7.2, and 9.6 mg/mL GlcNAc groups were 12, 12, 10, 10, and 10, respectively.

The monitoring period lasted about 6 days, during which the mice underwent the second and third acclimation treadmill runs at speeds of 10 and 15 m/min for 10 min on Day 3 and Day 5, respectively, during the lights‐on period, as illustrated in Figure 3A. Following the acclimation phase, a downhill treadmill run with a gradual increase in speed from 8 to 15 m/min over a total of 49 min at a 15° incline was performed. DVC analysis showed that the effects of 7.2 and 9.6 mg/mL GlcNAc in mice subjected to forced exercise lost their activity advantage, showing spontaneous home‐cage activity levels similar to the untreated control group (Figure 6A vs. Figure 7A). In contrast, mdx mice treated with 2.4 and 4.8 mg/mL GlcNAc maintained significantly higher spontaneous activity compared to the control group.

However, a supplementary high‐resolution analysis using the raw, unbinned data (0.25‐s intervals) offered further insight. In this analysis, which is more sensitive to brief or intermittent movements, all GlcNAc‐treated groups showed significantly higher activity than the untreated controls (*p* < 0.0001) (Figure S3A). The most significant beneficial effect on locomotor activity was observed in the 2.4 mg/mL group, followed by the 4.8 mg/mL group, while the 7.2 and 9.6 mg/mL groups showed a more modest but still significant benefit (Figure S3A). Taken together, these findings suggest that while the lower doses of GlcNAc (2.4 and 4.8 mg/mL) confer the most consistent and substantial preservation of function after exercise challenges, a degree of benefit is retained across all doses, particularly when analyzing fine‐scale movement patterns.

During the lights‐on period, only the 2.4 mg/mL GlcNAc‐treated group showed significantly higher locomotor activity, while the other groups displayed similar or slightly reduced activity relative to the control (Figure 7B). Notably, during the lights‐off period, mdx mice treated with 2.4 and 4.8 mg/mL GlcNAc showed significantly higher locomotor activity (Figure 7C), suggesting that these doses of GlcNAc positively impact functional resilience, supporting muscle movement even after repeated eccentric contractions.

The heat map shown in Figure 5B corresponds to the mdx mice subjected to treadmill running (Protocol 2). After the 49‐min treadmill run performed on Day 8 during the lights‐on period, the locomotor activity of all groups of mdx mice remained relatively low, even during the lights‐off period (Figure 5B, Day 8). This consistency likely reflects the muscle damage induced by the 49‐min treadmill run, as demonstrated in Figure 4. In Figure 7C, the locomotor activity before (Slope A) and after (Slope B) the 49‐min run was analyzed, and in all groups, the activity was statistically significantly reduced, suggesting the negative impact of repeated eccentric contractions. Cumulative locomotor activity index after the 49‐min run is shown separately in Figure 7D. Despite the muscle damage induced by downhill running, mice treated with 2.4 mg/mL retained significantly higher locomotor activity compared to the control group. A supplementary high‐resolution analysis using the raw, unbinned data during this period also shows consistently that the most significant preservation of post‐exercise locomotor activity was observed in the 2.4 mg/mL group (Figure S3B).

These results, combined with both the binned and high‐resolution locomotor activity data, show that GlcNAc treatment at all tested doses promotes spontaneous locomotor activity in mice subjected to the acclimation phase level of exercise (horizontal treadmill running) (Slope A). However, there appears to be a threshold of exercise intensity beyond which higher doses of GlcNAc (e.g., 4.8 mg/mL) become ineffective. Additionally, it is important to note that even though GlcNAc becomes ineffective beyond this threshold, it does not appear to have any harmful effects on *mdx* mice, as no significant impact on the mice's BW, survival, or muscle mass was found (Figure 3).

### 3.8 Effect of GlcNAc and Prednisolone on the Locomotor Activity of *mdx* Mice (Protocol 3)

**FIGURE 8:**
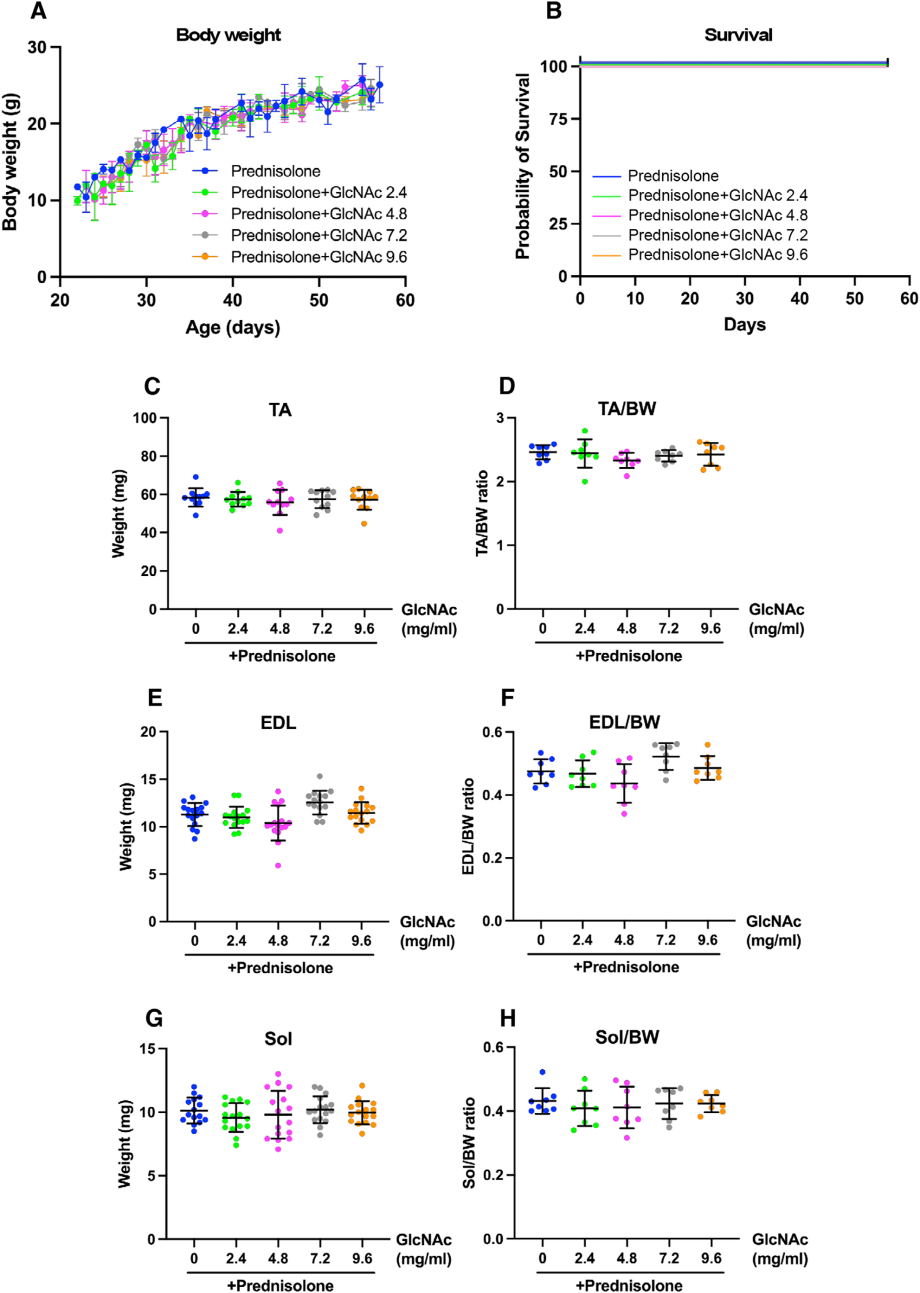
Impact of GlcNAc on BW, survival, and muscle mass in *mdx* Mice treated with Prednisolone (Protocol 3). GlcNAc (0, 2.4, 4.8, 7.2, and 9.6 mg/mL) was administered orally along with or without 1 mg/kg BW per day of prednisolone to *mdx* mice via voluntary intake through their drinking water. (A) Mice were treated for 35 days, during which their BW was regularly measured. (B) The survival rate of the mice was monitored throughout the study. For both (A and B), the number of mice used was 8. (C–H) After 35 days of treatment, the *mdx* mice were sacrificed, and the mass of the tibialis anterior (TA) (C), TA mass relative to BW (D), extensor digitorum longus (EDL) mass (E), EDL mass relative to BW (F), soleus (Sol) mass (G), and Sol mass relative to BW (H) were measured. For (C, E and G) the sample size was 16, and for (D, F, and H), the sample size was 8. Statistical analyses were performed using ordinary one‐way ANOVA with Tukey's post hoc test (A and C–I), and Mantel‐Cox test (B). No significant differences were observed. (A and C–I) Data represent means ± standard deviations.

**FIGURE 9:**
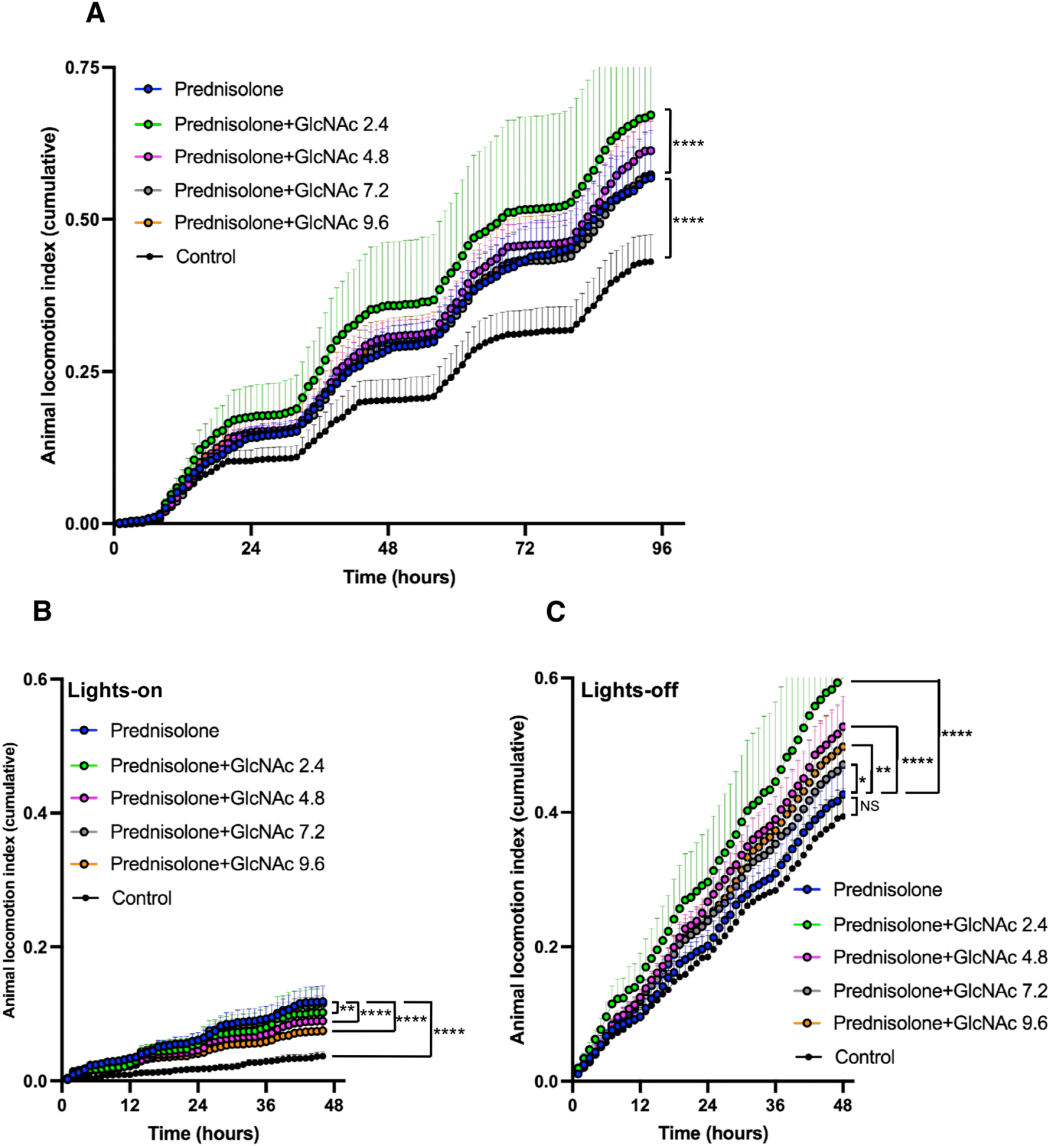
Effect of GlcNAc and Prednisolone on the Spontaneous Locomotor Activity Index of *mdx* Mice (Protocol 3). Mice were treated for 35 days and the locomotor activity was measured over the final 3.5 days before the end of the treatment period. The activity index was derived from raw data captured at 4 Hz, binned into 1‐s intervals, and then summed hourly for plotting. Error bands represent the SEM. For all panels, treatment groups are indicated by color as follows: Control non‐treated (black), Prednisolone alone (black outlined blue), Prednisolone+GlcNAc 2.4 (black outlined green), Prednisolone+GlcNAc 4.8 (black outlined magenta), Prednisolone+GlcNAc 7.2 (black outlined gray), and Prednisolone+GlcNAc 9.6 (black outlined orange). (A) Cumulative locomotor activity index during both lights‐on and lights‐off periods. (B) Cumulative locomotor activity index during the lights‐on period. (C) Cumulative locomotor activity index during the lights‐off period. Statistical analysis was performed using two‐way ANOVA with Dunnett's test. Asterisks indicate significance levels for two sets of comparisons: (1) the non‐treated control versus the prednisolone‐only group, and (2) the prednisolone‐only group versus the GlcNAc co‐treatment groups (*p < 0.05, **< 0.01, ***p < 0.001, and **p < 0.0001; NS, not significant). Note that all groups treated with prednisolone (both alone and in combination with GlcNAc) showed significantly higher activity than the non‐treated control group (*p* < 0.0001); for clarity, these comparisons are not marked on the figure. The number of mice used for Control, and for 2.4, 4.8, 7.2, and 9.6 mg/mL GlcNAc groups were 6, 8, 8, 8, and 8, respectively.

Prednisolone is commonly used in the intervention of DMD patients to suppress inflammation [10, 11, 12]. In Protocol 3, we also investigated how prednisolone influences the effects of GlcNAc. As shown in Figure 8, the addition of various doses of GlcNAc did not significantly affect BW gain trajectory over the course of the experiment (Figure 8A) and the overall survival rate (Figure 8B) when compared to treatment with prednisolone alone. At the study's endpoint, we also observed no significant effects of GlcNAc cotreatment on absolute muscle mass (Figure 8C,E,G) or the BW‐normalized mass (Figure 8D,F,H) of the TA, EDL, or Sol muscles. To determine the effects of prednisolone treatment itself, we then compared the data from steroid‐treated animals to that from non‐steroid‐treated cohorts (Figure S4). This comparison revealed that prednisolone treatment significantly reduced endpoint body weights (as shown in Figure S4A). This finding is consistent with previous reports that long‐term glucocorticoid use can promote muscle catabolism [46]. Critically, the muscle‐to‐body‐weight ratios showed no significant differences between the steroid and non‐steroid groups (Figure S4, panels for TA/BW, EDL/BW, Sol/BW). We then used the DVC system to evaluate the locomotor activity index under combination treatment with prednisolone and GlcNAc. As expected, prednisolone alone significantly increased the spontaneous locomotor activity of *mdx* mice compared to the non‐treated control group (Figure 9A). Based on the standard (binned) data analysis, only the 2.4 mg/mL GlcNAc combined with prednisolone showed a statistically significant increase in overall locomotor activity compared to prednisolone alone. However, a more sensitive, high‐resolution analysis of the raw (unbinned) data revealed a broader therapeutic effect. In this analysis, co‐treatment with GlcNAc at 2.4 and 4.8 mg/mL resulted in a highly significant increase in activity compared to prednisolone alone (*p* < 0.0001), while the 9.6 mg/mL dose also conferred a significant, albeit more modest, increase (*p* < 0.05) (Figure S5).

Further analysis of the locomotor activity index during the lights‐on period (Figure 9B) revealed that prednisolone alone significantly increased locomotor activity, whereas GlcNAc mitigated this increase. In contrast, during the lights‐off period, all doses of GlcNAc combined with prednisolone enhanced the locomotor activity of *mdx* mice compared to treatment with prednisolone alone (Figure 9C), suggesting GlcNAc has the capacity to further improve the muscle health of mice undergoing prednisolone treatment. Due to GlcNAc's counteracting effect on prednisolone during the lights‐on period and its promotion of activity during the lights‐off period, only the 2.4 mg/mL GlcNAc dose showed a significant effect when considering combined lights‐on and lights‐off data (Figure 9A). Prednisolone is known to have many side effects including insomnia [47], although there were few reports examining its effects on insomnia in *mdx* mice. As shown in Figure 9B, the significant increase in diurnal activity in mice treated with prednisolone appears to be associated with insomnia. These results suggest that GlcNAc may counteract this abnormal activity during the lights‐on period. Nevertheless, these results suggest that the combination of GlcNAc and prednisolone promotes nocturnal locomotor activity during the lights‐off period. Given that the combination treatment did not impact BW, survival, or muscle mass, compared to prednisolone alone (Figure 8), it is unlikely to produce adverse effects. Table 1 summarizes the results, showing that GlcNAc, particularly at 2.4 mg/mL, consistently promotes the locomotor activity of *mdx* mice.

**TABLE 1:** Summary of the results of spontaneous locomotor activity.

Treatment	Lights	Locomotor activity	Relative to control (fold)	Relative to prednisolone (fold)
Control	On and Off	0.20240		
GlcNAc 2.4	**On and Off**	**0.29610**	**1.46294**	
GlcNAc 4.8	On and Off	0.29400	1.45257	
GlcNAc 7.2	On and Off	0.28750	1.42045	
GlcNAc 9.6	On and Off	0.24430	1.20702	
Control	On	0.01860		
GlcNAc 2.4	**On**	**0.04060**	**2.18280**	
GlcNAc 4.8	On	0.05900	3.17204	
GlcNAc 7.2	On	0.05000	2.68817	
GlcNAc 9.6	On	0.03150	1.69355	
Control	Off	0.20070		
GlcNAc 2.4	**Off**	**0.27930**	**1.39163**	
GlcNAc 4.8	Off	0.25580	1.27454	
GlcNAc 7.2	Off	0.25800	1.28550	
GlcNAc 9.6	Off	0.23020	1.14699	
Control+Treadmill	On and Off	0.46640		
GlcNAc 2.4 + Treadmill	**On and Off**	**0.59020**	**1.26544**	
GlcNAc 4.8 + Treadmill	On and Off	0.56530	1.21205	
GlcNAc 7.8 + Treadmill	On and Off	0.46270	0.99207	
GlcNAc 9.6 + Treadmill	On and Off	0.46130	0.98907	
Control+Treadmill	On	0.11460		
GlcNAc 2.4 + Treadmill	**On**	**0.14080**	**1.22862**	
GlcNAc 4.8 + Treadmill	On	0.12100	1.05585	
GlcNAc 7.8 + Treadmill	On	0.09770	0.85253	
GlcNAc 9.6 + Treadmill	On	0.11620	1.01396	
Control+Treadmill	Off	0.36050		
GlcNAc 2.4 + Treadmill	**Off**	**0.46160**	**1.28044**	
GlcNAc 4.8 + Treadmill	Off	0.45500	1.26214	
GlcNAc 7.8 + Treadmill	Off	0.37510	1.04050	
GlcNAc 9.6 + Treadmill	Off	0.35400	0.98197	
Control	On and Off	0.20240		0.73360
Prednisolone	On and Off	0.27590	1.36314	
Prednisolone + GlcNAc 2.4	**On and Off**	**0.33500**	**1.65514**	**1.21421**
Prednisolone + GlcNAc 4.8	On and Off	0.29030	1.43429	1.05219
Prednisolone + GlcNAc 7.8	On and Off	0.27340	1.35079	0.99094
Prednisolone + GlcNAc 9.6	On and Off	0.27820	1.37451	1.00834
Control	On	0.01960		0.29878
Prednisolone	On	0.06560	3.34694	
Prednisolone + GlcNAc 2.4	**On**	**0.05680**	**2.89796**	**0.86585**
Predinisolone + GlcNAc 4.8	On	0.05020	2.56122	0.76524
Predinisolone + GlcNAc 7.8	On	0.06450	3.29082	0.98323
Predinisolone + GlcNAc 9.6	On	0.04310	2.19898	0.65701
Control	Off	0.20070		0.91854
Prednisolone	Off	0.21850	1.08869	
Prednisolone + GlcNAc 2.4	**Off**	**0.31540**	**1.57150**	**1.44348**
Prednisolone + GlcNAc 4.8	Off	0.27030	1.34679	1.23710
Prednisolone + GlcNAc 7.8	Off	0.24560	1.22372	1.12403
Prednisolone + GlcNAc 9.6	Off	0.25570	1.27404	1.17025

*Note:* Fold difference relative to Control (water) and prednisolone are calculated. Results obtained using 2.4 mg/mL GlcNAc were indicated by black and right blue bold characters.

## 4 Discussion

### 4.1 Oral GlcNAc as a Safe and Efficacious Promoter of Spontaneous Locomotor Activity

GlcNAc, an endogenous precursor of UDP‐GlcNAc, serves as a substrate for various N‐acetylglucosaminyltransferases. Importantly, its intracellular concentration is closely associated with the levels of acetyllactosamine‐rich N‐linked oligosaccharides, which are recognized by the oligosaccharide‐binding protein galectin‐3 [13, 14, 15, 18, 19, 20, 21, 22, 23, 24, 25, 26, 27, 48]. This association is particularly significant because the rate‐limiting enzyme for the biosynthesis of these oligosaccharides requires a high concentration of UDP‐GlcNAc [17, 49, 50]. Notably, the interaction between galectin‐3 and acetyllactosamine‐rich N‐linked oligosaccharides plays a critical role in regulating the lateral movement of various membrane proteins (dynamics), as well as mediating cell–cell and cell‐extracellular matrix interactions [19, 20, 21, 22, 23, 24]. On the cell surface, these oligosaccharides contribute not only to the formation of the glycocalyx, which extends 50–500 nm above the cell surface but also to determining cell characteristics by presenting specific concentrations and aggregations of acetyllactosamine residues [19, 20, 21, 22, 23, 24]. The glycocalyx is vital for facilitating flexible interactions that support dynamic cellular processes [51]. Altering the glycocalyx by increasing the synthesis of acetyllactosamine‐rich N‐linked oligosaccharides through GlcNAc supplementation can potentially influence cell dynamics governed by the glycocalyx [19, 20, 21, 22, 23, 24]. Our previous and recent research demonstrated that GlcNAc promotes myotube formation in C2C12 cells and primary myoblasts [13, 27], and intraperitoneal administration of GlcNAc in *mdx* mice enhances muscle force [13]. These findings suggest that GlcNAc changes in the nature of the glycocalyx in the muscles of *mdx* mice, although the full implications of these changes remain unclear. In this study, we demonstrated that orally administered GlcNAc confers significant functional benefits, most notably by promoting the spontaneous locomotor activity of *mdx* mice.

We tested the effects of different GlcNAc doses dissolved in the drinking water at the concentrations of 2.4, 4.8, 7.2, and 9.6 mg/mL, which were equivalent to 0.6, 1.2, 1.8, and 2.4 g/kg BW per day, respectively, based on the observed mice's daily water intake. These doses in mice are equivalent to doses of 72, 144, 216, and 288 mg/kg BW per day, respectively, in humans, based on the body surface area normalization method [35]. Notably, this dose range is comparable to that used in human clinical trials for other inflammatory conditions. For instance, studies for patients with inflammatory bowel disease demonstrated therapeutic benefit at 12 g GlcNAc per patient [36]. Similarly, a recent open‐label clinical study in patients with multiple sclerosis using 6 and 12 g/day for 4 weeks reported reduced markers of inflammation and neurodegeneration [37]. This study also confirmed the treatment's safety, with only mild and tolerable gastrointestinal side effects observed at the 12 g/day dose, but not at 6 g/day. In our study, the effective doses of 2.4 and 4.8 mg/mL (equivalent 72 and 144 mg/kg BW in humans) fall within this clinically relevant range and consistently exhibited therapeutic impacts, including improved spontaneous locomotor activity and reduced muscle damage. For comparison, our previous study found that a lower dose of 250 mg/kg BW of GlcNAc per day for intraperitoneal administration for 10 days also exhibited the mitigation of DMD progression, including reduced muscle damage [13]. Considering the established efficient absorption of orally administered GlcNAc [17, 31], it is possible that doses lower than 2.4 mg/mL (600 mg/kg BW) GlcNAc may be sufficient for therapeutic effects. Therefore, it is essential to determine the minimal and most optimal doses for the preparation of a future clinical study in individuals with DMD.

A key finding of this study is that oral administration of GlcNAc significantly promotes spontaneous locomotor activity in *mdx* mice, a quantitative indicator of the functional status. This promotion does not appear to be a result of adverse effects. Furthermore, the observed increase in activity is unlikely to be a non‐specific ‘sugar rush,’ as GlcNAc is not a primary substrate for rapid energy production via glycolysis and water consumption did not differ between groups. Our examination revealed no apparent impact of GlcNAc on BW, survival, or the mass of TA, EDL, and Sol muscles. This is consistent with previous preclinical chronic toxicity studies, which demonstrated that oral administration of GlcNAc 2.5 g/kg BW per day (equivalent to 0.6 g/kg BW per day in humans) for 52 weeks results in no adverse effects or histopathological changes on tissues [33, 34]. Furthermore, evidence for the safety of this dose range comes from human physiology itself. Breastfed infants are naturally exposed to high levels of GlcNAc: this free monosaccharide is released by the infant's gut microbiota as it breaks down the abundant oligosaccharides present in human milk, leading to an estimated daily consumption estimated at 100–300 mg/kg of body weight [38, 39, 52]. This natural exposure level is similar to the effective human equivalent doses identified in our study (72 and 144 mg/kg/day), strongly supporting the interpretation that our therapeutic doses fall within a physiologically safe and relevant range. While most of GlcNAc doses in this study promote locomotor activity to some degree, GlcNAc at 2.4 and 4.8 mg/mL showed a more pronounced effect than 7.8 and 9.6 mg/mL of GlcNAc, suggesting that an optimal therapeutic window likely exists.

In the study by Ryczko et al., healthy wild‐type mice were treated with GlcNAc immediately after weaning for 90 days, at concentrations ranging from 0 to 15 mg/mL [53]. BWs appeared to be higher in the groups receiving 5 and 15 mg/mL GlcNAc, starting around day 20 of administration, compared to lower‐dose and untreated groups, although statistical significance was not reported. In contrast, when adult healthy wild‐type mice (3 months old) fed a 9% high‐fat diet were treated with 0.5 mg/mL GlcNAc for 210 days, BWs were significantly higher (*p* < 0.05) starting from day 70 of treatment. In our study, mdx mice were treated with GlcNAc (0.6–9.6 mg/mL) for 35 days immediately after weaning while receiving a 6% fat diet. This condition is more comparable to the early‐life intervention reported by Ryczko et al. rather than the prolonged adult treatment. Thus, given the shorter duration of treatment in our study, it is possible that GlcNAc did not have sufficient time to influence overall body weight. Furthermore, in the *mdx* model—where continuous muscle damage and regeneration require sustained structural and metabolic repair—GlcNAc may be preferentially directed toward biosynthetic and reparative pathways. As a result, GlcNAc may be rapidly utilized for these tissue‐specific processes, preventing systemic accumulation and limiting its effect on overall growth. This interpretation is also supported by the observed age‐dependent decline in circulating GlcNAc levels (Figure S6) [54], which may suggest an increasing physiological demand during growth and pathology that may necessitate exogenous supplementation. Therefore, this initial characterization demonstrates that oral GlcNAc holds significant promise as a safe intervention for improving locomotor function, even if it does not concurrently promote systemic growth in the unique context of the dystrophic model.

### 4.2 GlcNAc Enhances Functional Resilience Rather Than Conferring Acute Muscle Protection

Our previous reports indicated that intraperitoneal GlcNAc administration improves ex vivo muscular force in *mdx* mice [13], suggesting that GlcNAc alters the quality of muscles. The promoted locomotor activity observed in orally GlcNAc‐treated mice is likely related to these qualitative changes, which may enhance the functional capacity of dystrophic muscle. Indeed, histological analysis of EDL muscles revealed a significant reduction in damage formation in mice treated with 4.8 mg/mL GlcNAc administration. However, the extent of damage varied significantly, with some damage appearing to have formed immediately before sacrifice and others over a time frame that allowed some regeneration. This variability made it challenging to assess whether other GlcNAc doses reduced muscular damage and/or improved overall muscle health. To eliminate such experimental ambiguity and directly address whether GlcNAc protects skeletal muscles from acute injury, we conducted a downhill treadmill run to induce muscular damage 1 day before sacrifice. Our results indicated that GlcNAc does not prevent damage formation induced by repeated eccentric contractions. This conclusion is supported by a key contrast in our results. While oral GlcNAc significantly reduced histological damage in non‐exercised mice (at the 4.8 mg/mL dose), it did not prevent damage formation following a high‐intensity downhill treadmill run. This aligns with our previous work showing that while intraperitoneally administrated GlcNAc improves the ex vivo specific force of dystrophic muscle, it offers no protection from a subsequent ex vivo repeated eccentric contraction challenge [13].

A major advantage of our experimental design is the use of the DVC system, which provides a stress‐free, longitudinal measure of spontaneous activity. This method quantitatively assesses overall muscle function and reveals a complex, dose‐dependent effect of GlcNAc when combined with exercise‐induced stress. Based on standard binned data, lower doses (2.4 and 4.8 mg/mL) were more effective at providing resilience against moderate sustained exercise. However, a more nuanced picture emerges from the high‐resolution, unbinned data, which showed that all GlcNAc doses significantly increased overall activity. This suggests that while a broad benefit to overall activity exists across all tested doses, a narrower therapeutic window—likely around 2.4–4.8 mg/mL—appears to be crucial for maximizing functional resilience under physical stress.

Given that muscle regeneration is a slow process requiring at least 10 days [55], this observed resilience is likely a consequence of the muscle being in a pre‐existing healthier state established by the long‐term actions of GlcNAc. Therefore, we propose that GlcNAc works through multiple beneficial mechanisms. These mechanisms likely include (1) promoting muscle fiber regeneration, consistent with our in vitro observations that GlcNAc promotes myogenesis [13, 27] and (2) exerting anti‐inflammatory effects similar to those observed in other inflammatory diseases [17, 32, 37, 56, 57]. Beyond its effects on muscle, the CNS may represent another target for GlcNAc in the dystrophic context. Recent studies in models of multiple sclerosis suggest that GlcNAc can promote the repair of myelin sheaths [37, 52]. This is particularly relevant as the lack of dystrophin is known to negatively impact the CNS, increasing neuronal susceptibility to damage and causing alterations in myelination within the *mdx* mouse brain [58]. Therefore, it is plausible that the effects of GlcNAc on the CNS—in addition to its potential effects on muscle regeneration and inflammation—contributed to the improvements we observed. Thus, GlcNAc may enhance spontaneous locomotor activity by acting on multiple organs and systems, not limited to skeletal muscle alone.

### 4.3 The Biphasic Dose–Response: A Multifactorial Hypothesis

An observation from our study is the biphasic dose–response of GlcNAc, where the most significant therapeutic effects were observed in the 2.4 or 4.8 mg/mL groups, while higher doses showed diminished benefits without causing apparent adverse effects. This non‐linear relationship suggests that the biological response to GlcNAc is tightly regulated. Considering GlcNAc is a naturally present monosaccharide, its metabolism is likely governed by several feedback mechanisms. We propose three plausible explanations for this phenomenon: (1) Activity‐Induced Muscle Stress: In the context of the *mdx* model, intermediate doses of GlcNAc may optimally improve muscle health, leading to increased spontaneous activity. However, at higher doses, a stronger initial therapeutic effect could lead to a more substantial increase in locomotor activity. Given the inherent fragility of dystrophic muscle, this heightened activity might, in turn, cause additional mechanical stress and secondary damage, ultimately offsetting some of the initial benefits. This scenario would be consistent with the observed plateau or reversal of therapeutic gains at the highest doses. (2) Metabolic Feedback Inhibition: At very high concentrations, the cellular pathways responsible for GlcNAc metabolism may become saturated. This could include the saturation of key enzymes like GlcNAc kinase or UDP‐GlcNAc pyrophosphorylase, limiting further increases in intracellular UDP‐GlcNAc levels. Furthermore, the regulatory networks governing N‐ and O‐glycosylation likely engage compensatory feedback mechanisms to prevent over‐modification of proteins under supraphysiological substrate conditions. These homeostatic mechanisms could blunt the glycosylation effects and thereby diminish the functional impact at higher doses. (3) Allosteric Regulation of the biosynthesis of lactosamine‐rich N‐Glycan: the role of GlcNAc in enhancing the synthesis of lactosamine‐rich N‐glycan, a process primarily regulated by the glycosyltransferases MGAT4 and MGAT5 has been well established [16, 24, 31, 49]. Recent works from the group led by Kizuka provide a direct mechanism for a biphasic response [15, 59, 60]: they demonstrated that MGAT4 activity is allosterically inhibited by its own product (highly branched lactosamine‐rich N‐glycans), while MGAT5 protein levels are controlled via proteolytic cleavage. This type of product‐induced feedback inhibition offers a molecular explanation for why simply increasing the substrate (GlcNAc) beyond a certain point would fail to produce a greater effect and could even lead to a diminished response.

### 4.4 GlcNAc Retains Efficacy and Provides Additive Benefit in Combination With Prednisolone

Given that corticosteroids like prednisolone are a cornerstone of the standard of care for DMD [10, 11, 12], we investigated the effect of co‐administering GlcNAc with prednisolone in *mdx* mice [10, 11, 12, 56, 59]. As expected, prednisolone alone promoted spontaneous locomotor activity in *mdx* mice. The addition of GlcNAc resulted in a complex, diurnal‐specific interaction. During the active (lights‐off) period, GlcNAc significantly increased nocturnal activity, suggesting a potential for additive therapeutic benefit. Conversely, during the inactive (lights‐on) period, GlcNAc reduced the abnormal diurnal hyperactivity induced by prednisolone, a response that may relate to known corticosteroid side effects such as insomnia [47]. This stabilizing diurnal effect contrasts with the pattern observed when GlcNAc was administered alone. In the GlcNAc‐only cohorts, we also saw an increase in activity during the lights‐on period. However, detailed temporal analysis revealed this was primarily confined to the first hour of the light phase. Therefore, rather than indicating a perturbed sleep cycle, we hypothesize this reflects enhanced stamina and resilience; the treated mice were less fatigued from their nocturnal activity and could sustain it for longer into the morning. These complex, context‐dependent effects on diurnal activity are intriguing. They raise the possibility of an interplay between GlcNAc's actions and the circadian clock system, which is known to be dysregulated in DMD [61, 62, 63]. This prompts several compelling questions for future studies. For instance, the effects could stem from GlcNAc's known ability to traverse the blood–brain barrier and influence CNS processes [37]. Alternatively, there may be an interaction with corticosteroid‐driven signaling, as these hormones are powerful regulators of circadian rhythm and can mitigate desynchrony at muscle injury sites [61, 64]. Ultimately, regardless of the precise mechanism, the observation that GlcNAc provides an additional therapeutic effect in the presence of a standard‐of‐care corticosteroid is highly relevant and underscores its potential for clinical application in DMD.

### 4.5 Therapeutic Promise as a Broadly Applicable Supportive Therapy

An important consideration for the therapeutic application of GlcNAc is its established pharmacokinetic profile and the functional benefits we observed. Orally administered GlcNAc is known to be rapidly absorbed with kinetics similar to glucose, but it also has a short circulatory half‐life as it is efficiently taken up by tissues [31]. It is also well established that cellular GlcNAc is quickly converted into its bioactive intracellular form, UDP‐GlcNAc, which serves as the direct substrate for the glycosylation pathways; unlike glucose, it is not readily catabolized for energy via glycolysis or the pentose phosphate pathway [65]. Therefore, the accumulation of UDP‐GlcNAc in muscle is likely a more relevant indicator of target engagement than systemic GlcNAc levels. A limitation of the present study is that we did not directly quantify these UDP‐GlcNAc levels in muscle. Future studies will be essential to establish a full pharmacokinetic/pharmacodynamic relationship by correlating tissue UDP‐GlcNAc concentrations with the degree of functional improvement. Nevertheless, the robust functional benefits demonstrated in this study provide strong in vivo evidence that our oral dosing regimen achieves a physiologically significant effect in the target tissues.

We recently demonstrated that GlcNAc can create an optimal environment for myogenesis by regulating myoblasts' coordinated flow and aligning myoblasts along the eventual shapes of regenerated myofibers in vitro [27]. Based on this finding together with the results present in this study, a plausible hypothesis is that the therapeutic benefits observed in our *mdx* mice are not due to the prevention of acute damage, but rather are mediated by multiple beneficial mechanisms. These likely include its potential to support a more effective muscle regeneration process (as suggested by our in vitro data) as well as its capacity to modulate chronic inflammation (consistent with its known anti‐inflammatory effects in other diseases) [13, 37, 66, 67]. Given that GlcNAc administration showed no adverse effects in our study, it holds promise as a safe supportive therapy. The observation that endogenous GlcNAc levels decrease with age in both wild‐type and *mdx* mice (Figure S5) [54] further suggests that supplementation could be beneficial in this chronic disease context. While the optimal human dose remains to be determined, this current study suggests that GlcNAc has the potential to improve functional status, both alone and in combination with corticosteroids. Finally, because this monosaccharide‐based therapy appears to act through mechanisms distinct from most existing and pipeline treatments for DMD—such as gene correction or exon skipping—it represents a promising and broadly applicable adjunctive therapy to complement emerging treatments and improve the standard of care.

## Author Contributions

M.S.S. and S.S. designed the study, conducted experiments, and contributed to the manuscript's writing. G.S.‐P., M.F., and A.R. conducted experiments and analyzed H&E staining data.

## Conflicts of Interest

M.S.S., A.R., and S.S. are named as inventors on a patent for the use of GlcNAc for the treatment of muscular disorders.

## Supporting information


**Figure S1:** fsb271013‐sup‐0001‐FigureS1.pdf.

**Figure S2:** Effect of GlcNAc on the Spontaneous Locomotor Activity of mdx Mice (Protocol 3) (Analysis of Unbinned 4 Hz data). (A) Mice were treated with or without GlcNAc for 35 days, and locomotor activity was measured over the final 3.5 days before the end of the housing period. Cumulative locomotor activity index during both lights‐on and lights‐off periods, as indicated. The activity index was derived from raw data captured at a frequency of 4 Hz (every 0.25 s) and then further summed into 1‐h periods for plotting. (B) Cumulative distance is plotted over time. Error bands represent the SEM. Treatment groups are indicated by color as follows: Control non‐treated (blue), GlcNAc 2.4 (green), GlcNAc 4.8 (magenta), GlcNAc 7.2 (grey), and GlcNAc 9.6 (orange). Statistical analysis was performed using two‐way ANOVA with Dunnett's test. Significance levels are indicated as *p < 0.05, **p < 0.01 and ****p < 0.0001. The number of mice used for Control (0, untreated), and for 2.4, 4.8, 7.2, and 9.6 mg/mL GlcNAc groups were 4, 5, 6, 5, and 6, respectively.

**Figure S3:** fsb271013‐sup‐0003‐FigureS3.pdf.

**Figure S4:** fsb271013‐sup‐0004‐FigureS4.pdf.

**Figure S5:** fsb271013‐sup‐0005‐FigureS5.pdf.

**Figure S6:** fsb271013‐sup‐0006‐FigureS6.pdf.

## Data Availability

All data are included in the article and/or supporting information. The software was deposited to GitHub; DOI https://doi.org/10.5281/zenodo.12988726. Any questions regarding data availability can be directed to the corresponding author.
