# Supplementary material for: Improvement of Spontaneous Locomotor Activity in a Murine Model of Duchenne Muscular Dystrophy by N‐Acetylglucosamine Alone and in Combination With Prednisolone
Source: FASEB J. 2025 Sep 15;39(18):e71013. doi: 10.1096/fj.202500196R (PMC12434798; doi:10.1096/fj.202500196R)
Supplement: Supplementary file 1 — Figure S1: fsb271013‐sup‐0001‐FigureS1.pdf. [file FSB2-39-e71013-s002.pdf]

Sup.Fig. 1

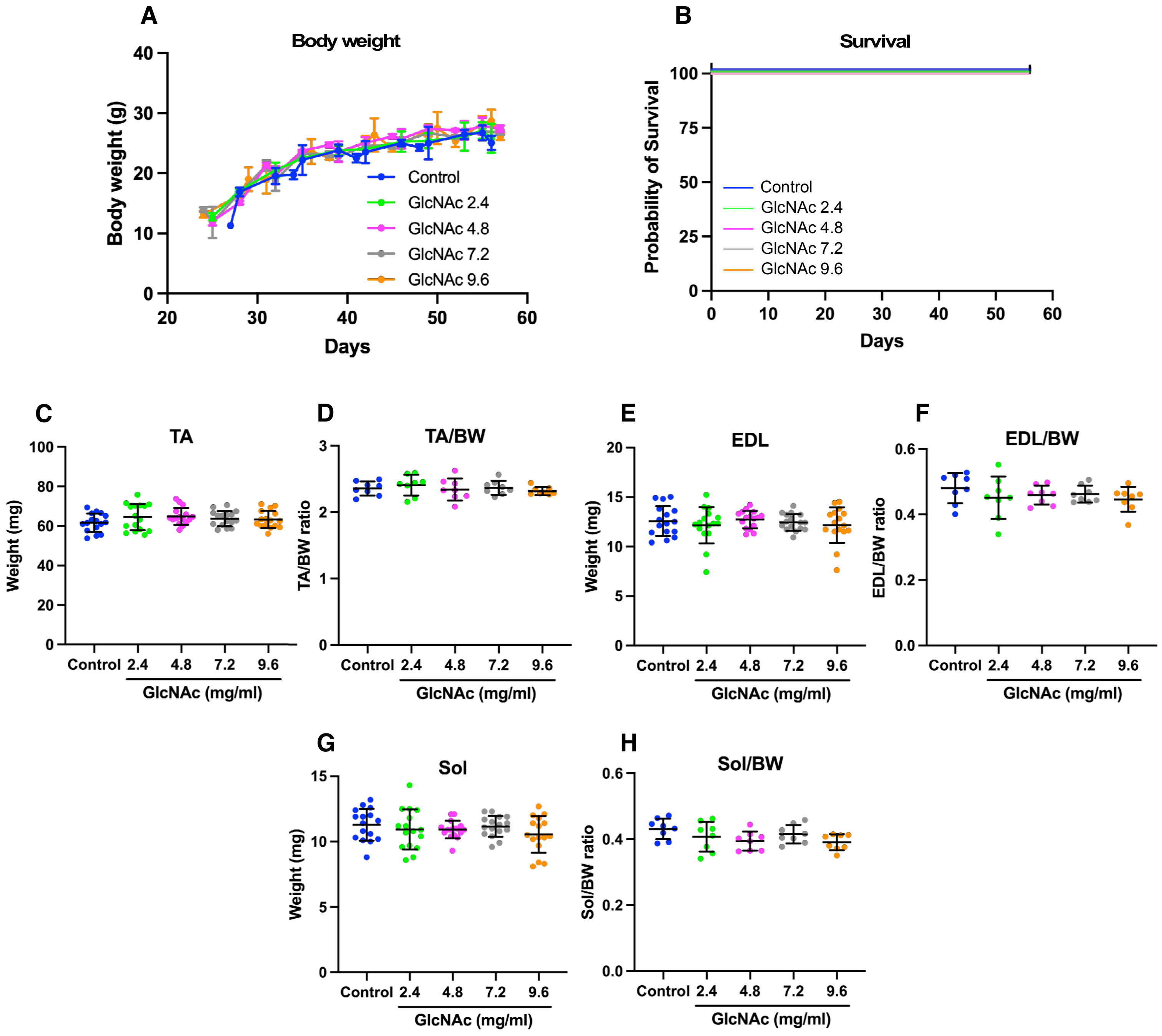

Supplementary Fig. 1. Analysis of the effect of GlcNAc on BW, survival, and muscle mass in *mdx* mice (Protocol 3-the groups without prednisolone treatment)

GlcNAc (2.4, 4.8, 7.2, and 9.6 mg/ml) was administered orally via voluntary intake through drinking water to mdx mice. Mice in the control group were provided with standard drinking water that did not contain any GlcNAc. **A.** Mice were treated for 35 days, during which their BW was regularly measured. **B.** The survival rate of the mice was monitored throughout the study. For both **A** and **B**, the number of mice used was 8. **C-H.** After 35 days of treatment, the *mdx* mice were sacrificed, and the mass of the TA (**C**), TA mass relative to BW (**D**), EDL mass (**E**), EDL mass relative to BW (**F**), Sol mass (**G**), and Sol mass relative to BW (**H**) were measured. For **C, E,** and **G**, the sample size was 16, and for **D, F,** and **H,** the sample size was 8. Statistical analyses were performed using ordinary one-way ANOVA with Tukey's post-hoc test (**A**, and **C-I), and** Mantel-Cox test (**B**). No significant differences were observed. **A**, and **C-I**. Data represent means ± standard deviations.

Sato et al.
