# Supplementary material for: Improvement of Spontaneous Locomotor Activity in a Murine Model of Duchenne Muscular Dystrophy by N‐Acetylglucosamine Alone and in Combination With Prednisolone
Source: FASEB J. 2025 Sep 15;39(18):e71013. doi: 10.1096/fj.202500196R (PMC12434798; doi:10.1096/fj.202500196R)
Supplement: Supplementary file 2 — Figure S2: Effect of GlcNAc on the Spontaneous Locomotor Activity of mdx Mice (Protocol 3) (Analysis of Unbinned 4 Hz data). (A) Mice were treated with or without GlcNAc for 35 days, and locomotor activity was measured over the final 3.5 days before the end of the housing period. Cumulative locomotor activity index during both lights‐on and lights‐off periods, as indicated. The activity index was derived from raw data captured at a frequency of 4 Hz (every 0.25 s) and then further summed into 1‐h periods for plotting. (B) Cumulative distance is plotted over time. Error bands represent the SEM. Treatment groups are indicated by color as follows: Control non‐treated (blue), GlcNAc 2.4 (green), GlcNAc 4.8 (magenta), GlcNAc 7.2 (grey), and GlcNAc 9.6 (orange). Statistical analysis was performed using two‐way ANOVA with Dunnett's test. Significance levels are indicated as *p < 0.05, **p < 0.01 and ****p < 0.0001. The number of mice used for Control (0, untreated), and for 2.4, 4.8, 7.2, and 9.6 mg/mL GlcNAc groups were 4, 5, 6, 5, and 6, respectively. [file FSB2-39-e71013-s001.pdf]

Sup.Fig. 2

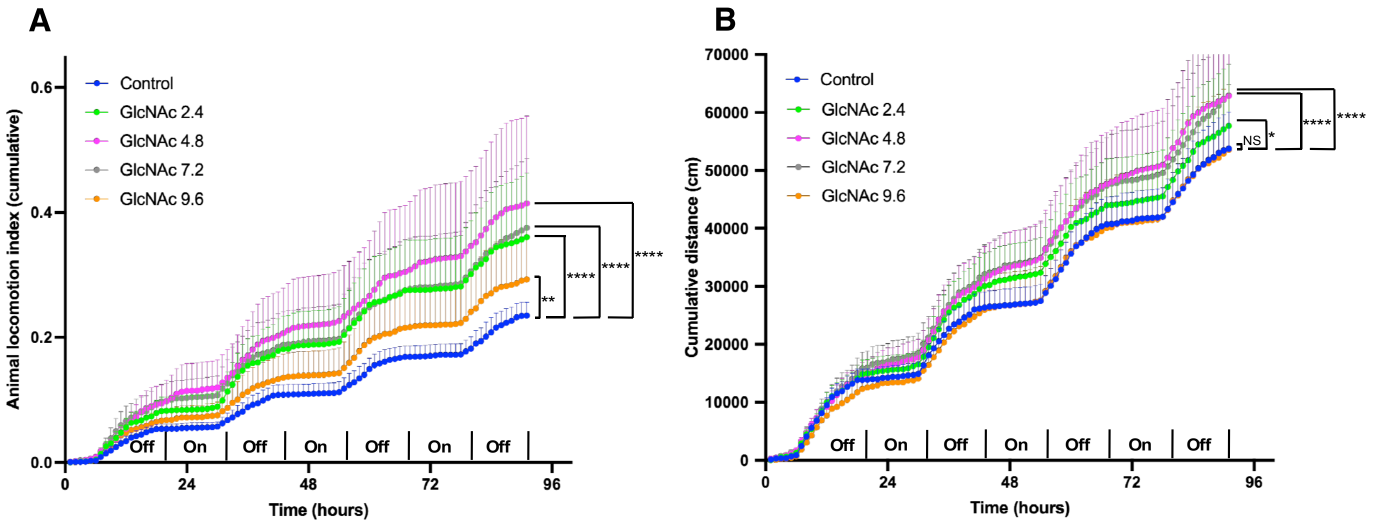

Supplementary Fig. 2. Effect of GlcNAc on the Spontaneous Locomotor Activity of *mdx* Mice (Protocol 3) (Analysis of Unbinned 4 Hz data)
A. Mice were treated with or without GlcNAc for 35 days, and locomotor activity was measured over the final 3.5 days before the end of the housing period. Cumulative locomotor activity index during both lights-on and lights-off periods, as indicated. The activity index was derived from raw data captured at a frequency of 4 Hz (every 0.25 seconds) and then further summed into 1-hour periods for plotting. **B**. Cumulative distance is plotted over time. Error bands represent the SEM. Treatment groups are indicated by color as follows: Control non-treated (blue), GlcNAc 2.4 (green), GlcNAc 4.8 (magenta), GlcNAc 7.2 (grey), and GlcNAc 9.6 (orange). Statistical analysis was performed using two-way ANOVA with Dunnett's test. Significance levels are indicated as * P<0.05, ** P <0.01 and ****P < 0.0001. The number of mice used for Control (0, untreated), and for 2.4, 4.8, 7.2, and 9.6 mg/ml GlcNAc groups were 4, 5, 6, 5, and 6, respectively.
