# Supplementary material for: Improvement of Spontaneous Locomotor Activity in a Murine Model of Duchenne Muscular Dystrophy by N‐Acetylglucosamine Alone and in Combination With Prednisolone
Source: FASEB J. 2025 Sep 15;39(18):e71013. doi: 10.1096/fj.202500196R (PMC12434798; doi:10.1096/fj.202500196R)
Supplement: Supplementary file 3 — Figure S3: fsb271013‐sup‐0003‐FigureS3.pdf. [file FSB2-39-e71013-s005.pdf]

Sup.Fig.3

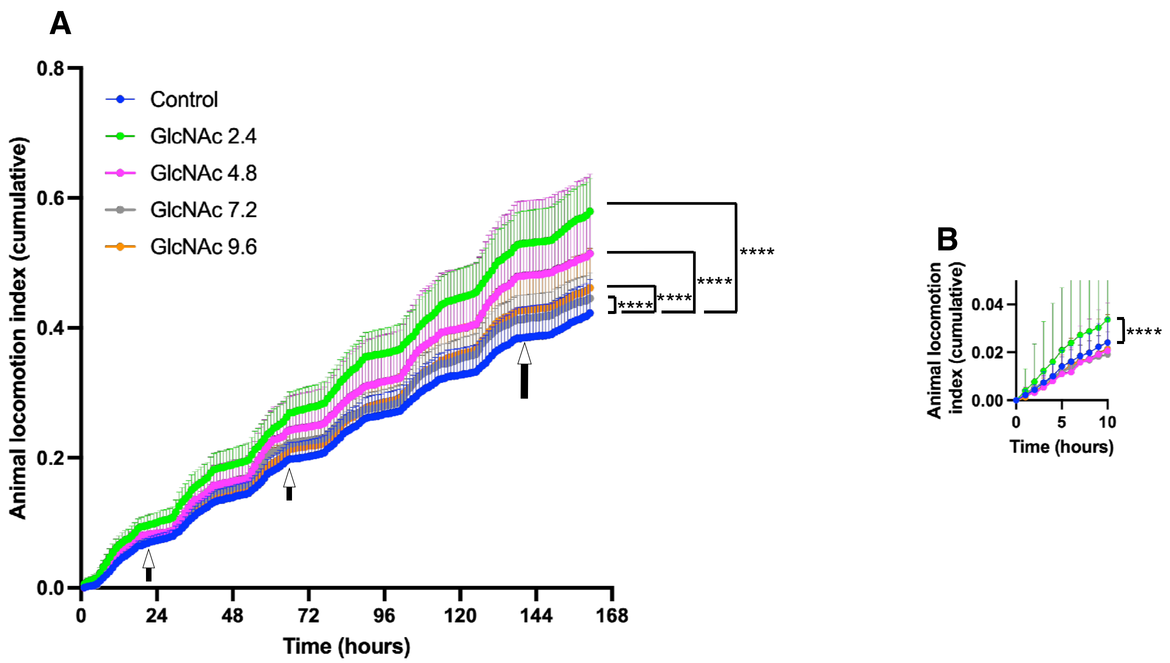

Supplementary Fig. 3. Effect of GlcNAc on the Spontaneous Locomotor Activity Index of *mdx* Mice Subjected to Treadmill Running (Protocol 2) (Analysis of Unbinned 4 Hz data)

Mice were treated with or without GlcNAc for 35 days. In the last week of the treatment, mice were subject to treadmill running according to the schedule illustrated in Fig. 3A. Locomotor activity was measured over the final 6.5 days before the mice were sacrificed. Error bands represent the SEM. For all panels, treatment groups are indicated by color as follows: Control non-treated (blue), GlcNAc 2.4 (green), GlcNAc 4.8 (magenta), GlcNAc 7.2 (grey), and GlcNAc 9.6 (orange). **A.** Cumulative locomotor activity index during both lights-on and lights-off periods. The first and second small arrows correspond to the acclimation treadmill runs performed on Day 3 and Day 5, respectively (Fig. 3A). The large arrow indicates when the 49-minute treadmill run with a 15-degree downhill incline, gradually increasing in speed from 8 to 15 m/min, was conducted. **B.** Cumulative locomotor activity index after the 49-minute treadmill run. Statistical analysis was performed using two-way ANOVA with Dunnett's test for A and two-way ANOVA with Tukey's test for B. Significance levels are indicated as ****$P < 0.0001$. The number of mice used in the Control group and the 2.4, 4.8, 7.2, and 9.6 mg/ml GlcNAc groups were 12, 12, 10, 10, and 10, respectively.
