# Supplementary material for: Improvement of Spontaneous Locomotor Activity in a Murine Model of Duchenne Muscular Dystrophy by N‐Acetylglucosamine Alone and in Combination With Prednisolone
Source: FASEB J. 2025 Sep 15;39(18):e71013. doi: 10.1096/fj.202500196R (PMC12434798; doi:10.1096/fj.202500196R)
Supplement: Supplementary file 4 — Figure S4: fsb271013‐sup‐0004‐FigureS4.pdf. [file FSB2-39-e71013-s006.pdf]

Sup.Fig. 4

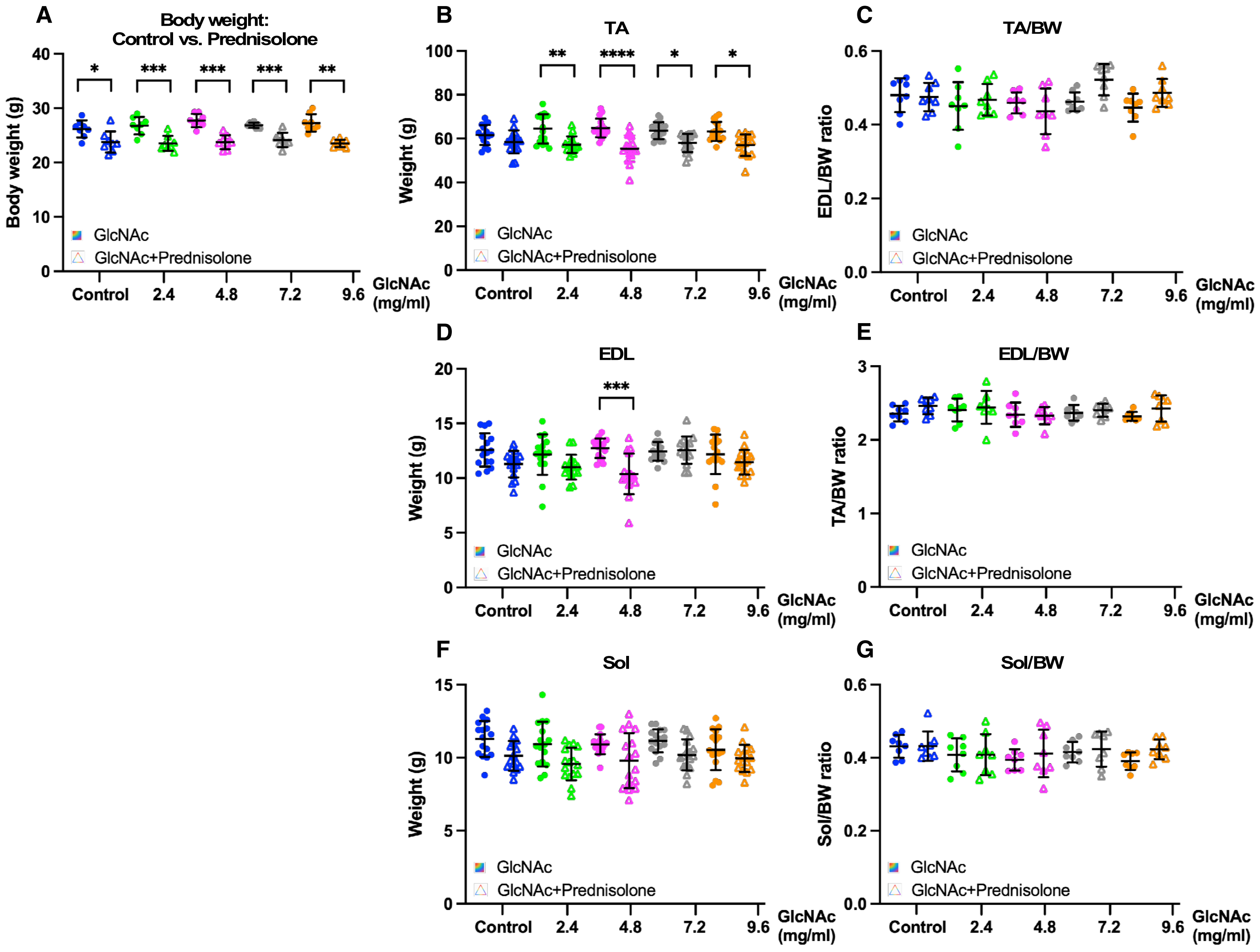

Supplementary Fig. 4 . Impact of GlcNAc on BW, survival, and muscle mass in *mdx* Mice treated with Prednisolone (Protocol 3)

GlcNAc (0, 2.4, 4.8, 7.2, and 9.6 mg/ml) was administered orally along with or without 1 mg/kg BW per day of prednisolone to *mdx* mice via voluntary intake through their drinking water. **A.** BW just before sacrifice. **B-G.** After 35 days of treatment, the *mdx* mice were sacrificed, and the mass of the tibialis anterior (TA) (**B**), TA mass relative to BW (**C**), extensor digitorum longus (EDL) mass (**D**), EDL mass relative to BW (**E**), soleus (Sol) mass (**F**), and Sol mass relative to BW (**G**) were measured. For **B, D** and **F**, the sample size was 16, and for **C, E** and **G,** the sample size was 8. Statistical analyses were performed using ordinary one-way ANOVA with Tukey's post-hoc test (**C** and **D-I**). Data represent means ± standard deviations.
