# Supplementary material for: Improvement of Spontaneous Locomotor Activity in a Murine Model of Duchenne Muscular Dystrophy by N‐Acetylglucosamine Alone and in Combination With Prednisolone
Source: FASEB J. 2025 Sep 15;39(18):e71013. doi: 10.1096/fj.202500196R (PMC12434798; doi:10.1096/fj.202500196R)
Supplement: Supplementary file 5 — Figure S5: fsb271013‐sup‐0005‐FigureS5.pdf. [file FSB2-39-e71013-s003.pdf]

Sup.Fig. 5

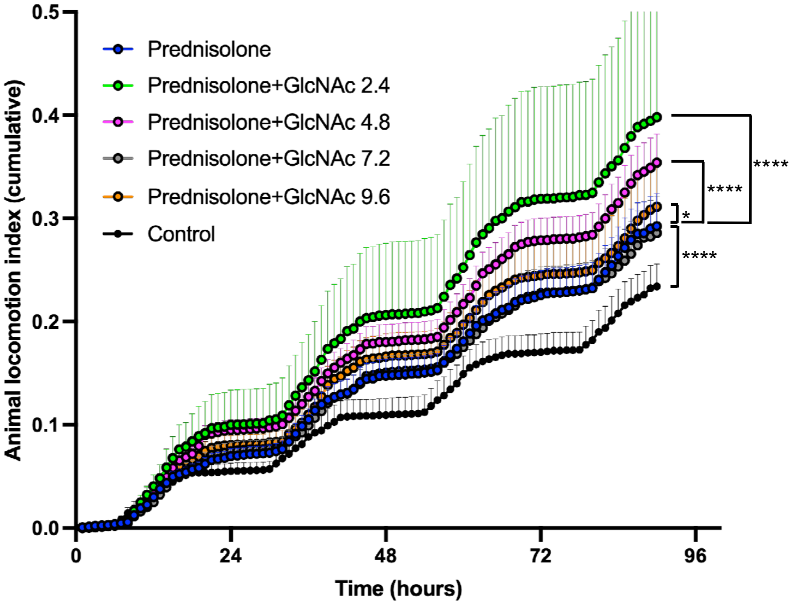

Supplementary Fig. 5. Effect of GlcNAc and Prednisolone on the Spontaneous Locomotor Activity Index of *mdx* Mice (Protocol 3) (Analysis of Unbinned 4 Hz dat)

Mice were treated for 35 days and the locomotor activity was measured over the final 3.5 days before the end of the treatment period. Cumulative locomotor activity index during both lights-on and lights-off periods. Error bands represent the SEM. Treatment groups are indicated by color as follows: Control non-treated (black), Prednisolone alone (black outlined blue), Prednisolone+GlcNAc 2.4 (black outlined green), Prednisolone+GlcNAc 4.8 (black outlined magenta), Prednisolone+GlcNAc 7.2 (black outlined grey), and Prednisolone+GlcNAc 9.6 (black outlined orange). Statistical analysis was performed using two-way ANOVA with Dunnett's test. Asterisks indicate significance levels for two sets of comparisons: (1) the non-treated control versus the prednisolone-only group, and (2) the prednisolone-only group versus the GlcNAc co-treatment groups (*P < 0.05, and **P < 0.0001). Note that all groups treated with prednisolone (both alone and in combination with GlcNAc) showed significantly higher activity than the non-treated control group (P < 0.0001); for clarity, these comparisons are not marked on the figure. The number of mice used for Control, and for 2.4, 4.8, 7.2, and 9.6 mg/ml GlcNAc groups were 6, 8, 8, 8, and 8, respectively.
