# Supplementary material for: Improvement of Spontaneous Locomotor Activity in a Murine Model of Duchenne Muscular Dystrophy by N‐Acetylglucosamine Alone and in Combination With Prednisolone
Source: FASEB J. 2025 Sep 15;39(18):e71013. doi: 10.1096/fj.202500196R (PMC12434798; doi:10.1096/fj.202500196R)
Supplement: Supplementary file 6 — Figure S6: fsb271013‐sup‐0006‐FigureS6.pdf. [file FSB2-39-e71013-s004.pdf]

Sup. Fig. 6

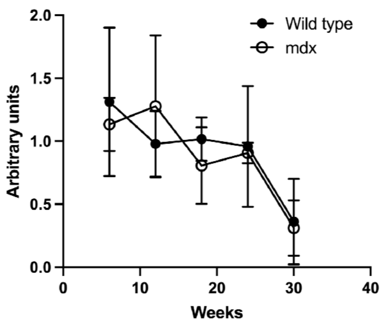

Supplementary Fig. 6. Reduced Concentration of GlcNAc in the Blood of *mdx* and wild-type Mice with AgingAge-related reduction of the concentrations of GlcNAc in the blood of wild-type and *mdx* mice. Data were extracted from Supplementary Table S3 provided by Tsonoka et al.[65] The data were reanalyzed and plotted to illustrate the decline in blood GlcNAc concentration along with aging. Data represent means ± standard deviations.
